# Transcriptomic and macroscopic architectures of intersubject functional variability in human brain white-matter

**DOI:** 10.1038/s42003-021-02952-y

**Published:** 2021-12-20

**Authors:** Jiao Li, Guo-Rong Wu, Bing Li, Feiyang Fan, Xiaopeng Zhao, Yao Meng, Peng Zhong, Siqi Yang, Bharat B. Biswal, Huafu Chen, Wei Liao

**Affiliations:** 1 The Clinical Hospital of Chengdu Brain Science Institute, MOE Key Laboratory for Neuroinformation, University of Electronic Science and Technology of China, Chengdu, 611731 P.R. China; 2 The Center of Psychosomatic Medicine, Sichuan Provincial Center for Mental Health, Sichuan Provincial People’s Hospital, University of Electronic Science and Technology of China, Chengdu, 611731 P.R. China; 3 School of Life Science and Technology, Center for Information in BioMedicine, University of Electronic Science and Technology of China, Chengdu, 611731 P.R. China; 4 Key Laboratory of Cognition and Personality, Faculty of Psychology, Southwest University, Chongqing, 400715 P.R. China; 5 Department of Biomedical Engineering, New Jersey Institute of Technology, Newark, NJ 07103 USA

**Keywords:** Neural circuits, Gene expression

## Abstract

Intersubject variability is a fundamental characteristic of brain organizations, and not just “noise”. Although intrinsic functional connectivity (FC) is unique to each individual and varies across brain gray-matter, the underlying mechanisms of intersubject functional variability in white-matter (WM) remain unknown. This study identified WMFC variabilities and determined the genetic basis and macroscale imaging in 45 healthy subjects. The functional localization pattern of intersubject variability across WM is heterogeneous, with most variability observed in the heteromodal cortex. The variabilities of heteromodal regions in expression profiles of genes are related to neuronal cells, involved in synapse-related and glutamic pathways, and associated with psychiatric disorders. In contrast, genes overexpressed in unimodal regions are mostly expressed in glial cells and were related to neurological diseases. Macroscopic variability recapitulates the functional and structural specializations and behavioral phenotypes. Together, our results provide clues to intersubject variabilities of the WMFC with convergent transcriptomic and cellular signatures, which relate to macroscale brain specialization.

## Abstract (web summary)

Jiao Li et al. evaluate the genetic basis underlying white matter functional connectivity variabilities in healthy human subjects. Their results shed light on how transcriptomic and cellular signatures converge to relate to macroscale brain specialization.

## Introduction

Blood-oxygenation-level-dependent-functional magnetic resonance imaging (BOLD-fMRI) has become the method of choice for evaluating the coherent signal fluctuations across the brain, because it has high spatial and temporal resolutions and is noninvasive^1–3^. This intrinsically inter-regional functional connectivity (FC) architecture is unique among individuals^4–7^ and reflects cognitive or population variabilities^8,9^. A higher intersubject variability of gray-matter (GM) FC reliably exists in higher-order cognitive networks, and lower intersubject variability exists in lower-order perceptual networks, suggesting intersubject variability gradients^10–12^. There is intersubject variability in cortical organization, even after accurate brain structure alignment of brain functions to structures^8^. Although white-matter (WM) comprises half of the human brain, its variabilities in connectomic organization across individuals have been relatively unexplored^13,14^.

Brain WM signals measured using BOLD-fMRI provide functional information about intrinsic activity and can be used to characterize its connectivity^15,16^. WM functional networks that are aligned and interact across long-distance WM tracts^17^ and functional organization of human corpus callosum that is closely adjacent to resting-state GM networks^18^, have highlighted the functional roles of WM. In a recent study, WM functional topography (e.g., small-wordless and nonrandom modularity) was computed and its reliability and reproducibility demonstrated, suggesting that WM functional connectivity (WMFC) cannot be simply attributed to noise^19^. In addition, it has been reported that intersubject variation in WMFC can be used to estimate individual general fluid intelligence^20^. Therefore, WMFC is unlikely to be generated by noise^21^, and may provide important information that contributes to the understanding of the underlying mechanisms of individual variabilities in cognition and behavior.

The function of WM often overlaps with and is constrained by the physical substrate’s known anatomical pathways/microstructures^22^. WM microstructure is a potential predictor of intersubject variations, because it is under strong genetic control. The WM associated genes regulate pathways involved in brain disease pathogenesis and neurodevelopmental processes^23^. Several genome-wide association studies^24–27^ have been conducted to identify loci associated with interindividual variations in WM microstructure, but these studies have been impeded by spatial specificities, which have been the major limitation^28^. In this study, we used an approach for combining neuroimaging and spatial patterned gene expression to investigate the molecular mechanisms of individual variabilities in FC architecture^29,30^. The Allen Human Brain Atlas (AHBA) microarray dataset was used to identify transcriptomes associated with human neuroimaging with multi-modal evidence, suggesting a link between conserved gene expression and relevant functional circuitries^31–33^. To the best of our knowledge, no study has previously characterized intersubject variabilities in WMFC-related genes using transcriptome-neuroimaging association analyses, therefore, the genetic influences on intersubject variabilities in WMFC are, thus far, unknown.

**Fig. 1:**
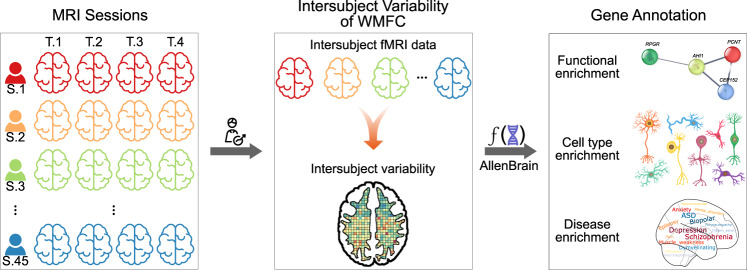
Data generation and analysis pipeline. This study was performed using neuroimaging and six AHBA transcriptomic data. Gene expression maps were extracted from NeuroSynth (https://www.neurosynth.org). Neuroimaging data were collected from 45 subjects who underwent four sessions within approciately 6 months. After establishing the relationship between intersubject variability in white-matter functional connectivity and gene expression, the variability-related highly-ranked gene set was identified. Enrichment analysis of the gene set was used to identify the functions of these genes. The identified intersubject variability-related genes were annotated by functional, cell types, and disease enrichments.

Our objective was to identify the intersubject variability of WMFC-related transcriptional architecture among individuals. Considering intersubject variabilities in functional organizations, researchers have further characterized brain networks responsible for the etiologies of disorders^34–36^. More recently, Sun et al.^37^ have reported alterations of intersubject variabilities of the FC in schizophrenia, suggesting potential implications for understanding the high clinical heterogeneity of this disorder. We therefore hypothesized that this information would increase our understanding of the genetic causess of intersubject variabilities of WMFC in healthy subjects, and may be associated with psychiatric and neurological brain disorders. We collected resting-state BOLD-fMRI data on 45 healthy subjects, and each scanned four times over ~6 months. This unique data set allowed us to assess the interindividual variabilities in WMFC, which controlled intrasubject variabilities^10,38^. We determined the following: (i) whether intersubject variabilities in WMFC were hierarchically organized, and whether the degree of variability varied from unimodal to heteromodal association networks; (ii) how individual variabilities in WMFC were affected by the transcriptome and specific cell types; (iii) whether clinical significance could be calculated by the overlap between intersubject variability-related genes and those involved in diseases; and (iv) whether the intersubject variability was related with known macroscale indices, including structural and functional organizations. Our findings will facilitate the study of both the spatial distribution of intersubject variabilities in WMFC and the multiscale associations in functional variabilities in brain WM across subjects. The data generation and analysis schematic are shown in Fig. 1.

## Results

### Study design and image quality assessment

The current work used a hybrid design (i.e., within + between design) in which scans were repeated one or more times on the same day and across one or more sessions^39^. All subjects participated in four sessions (each scanning included two runs) over ~6 months (Supplementary Result 1 and Supplementary Fig. 1). To measure the quality of the resting-state BOLD-fMRI data, we calculated a series of common image quality metrics using MRIQC^40^. Data were first visually inspected for the whole brain field of view coverage, signal artifacts, and proper alignment to the corresponding anatomical images. The image quality metrics for the spatial information included Entropy Focus Criterion, Foreground to Background Energy Ratio, Signal-to-noise ratio (SNR). The image quality metrics for the temporal information included Temporal SNR (tSNR) and Mean Fractional Displacement (mean FD; Supplementary Result 2 and Supplementary Fig. 2).

### Nonuniform distribution of intersubject variability

**Fig. 2:**
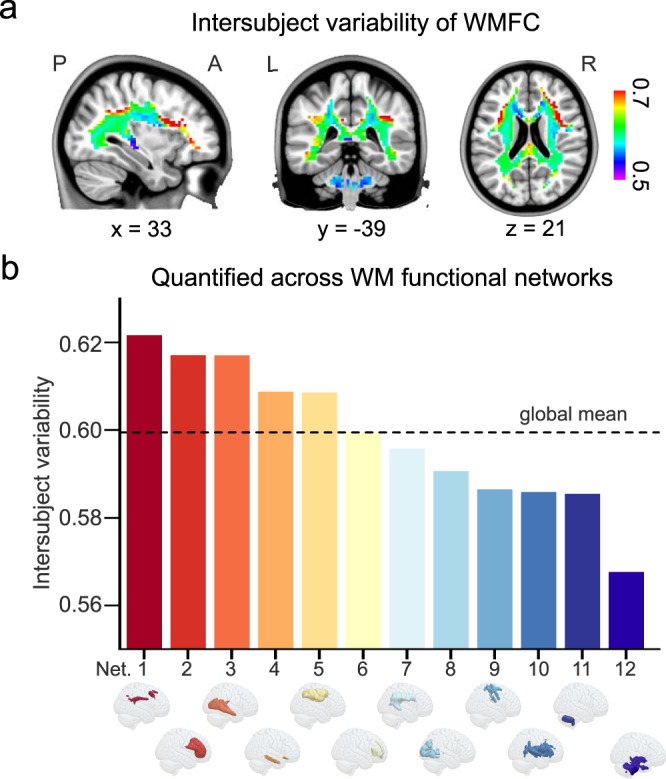
Nonuniform distribution of intersubject variability of white-matter functional connectivity (WMFC). **a** Variability distribution across voxels. Intersubject variability was quantified at each white-matter (WM) voxel across subjects after correcting for intrasubject variability and technical noise. **b** Intersubject variation in WMFC at the network level based on previous parcellation of the WM into 12 functional networks^17^. Network (Net) 1, frontoparietal control network; Net 2, deep frontal network; Net 3, inferior longitudinal fasciculus system; Net 4, temporal-orbitofrontal network; Net 5, dorsal frontoparietal network; Net 6, forceps minor system; Net 7, superior longitudinal fasciculus system; Net 8, visual network; Net 9 & 10, sensorimotor networks; and Net 11 & 12, cerebellum networks. Source data provided as Source Data file.

To study the organizational patterns of WMFC, we first estimated voxel-wise interindividual variabilities in the brain WM after controlling for intrasubject variability and technical noise (Supplementary Result 3 and Supplementary Fig. 3). We found that FC interindividual variability exhibited a nonuniform spatial distribution across brain WM voxels (Fig. 2a).

We further assessed the variabilities of the WMFC organization within each of the specific brain WM functional networks^17^. The hierarchy of the functional variations in WM across 12 specific networks was similar to that obtained from the prior gray-matter FC results^10–12,41^. Specifically, the frontoparietal control and default mode networks showed high levels of functional variability, while the sensorimotor, and visual networks exhibited low interindividual variations (Fig. 2b). In contrast to the gray-matter FC variations, the lowest variation was found in the visual and sensorimotor networks, where the cerebellum network showed the lowest level of functional variability in WM.

### The gene expression patterns associated with intersubject variability of WMFC

**Fig. 3:**
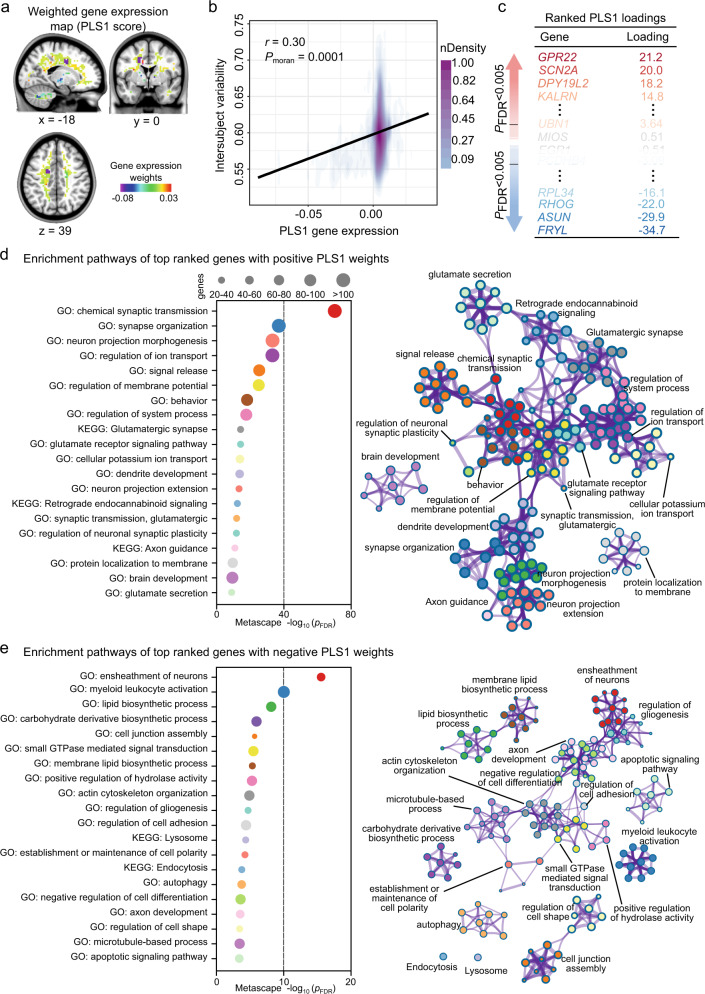
Transcriptomic decoding of the intersubject variability of white-matter functional connectivity (WMFC). **a** Weighted gene expression map of the first component of partial least squares (PLS1). The combination of genes, the most explained variance of intersubject variability in WMFC, was obtained by the PLS correlation method. **b** Scatterplot of the relationship between PLS1 scores and intersubject variability in WMFC. **c** Ranked PLS1 loading. Genes that were strongly positively weighted on PLS1 (e.g., *GPR22*) correlated positively with intersubject variability, whereas genes that were strongly negatively weighted on PLS1 (e.g., *FRYL*) correlated negatively with intersubject variability. Functional enrichment analysis of top ranked genes (from **c**) with PLS1 + (**d**) and PLS1− (**e**) weights. All terms were retained after *P* < 0.05, false discovery ratio-corrected. nDensity, density estimate, scaled to a maximum of 1. Source data provided as Source Data file.

We used the AHBA, a whole-brain transcriptomic dataset, to obtain patterns of gene expression in the brain for examining the transcriptomes associated with WMFC intersubject variability (Supplementary Result 4 and Supplementary Table 1). We aligned the nonuniform distribution map to the atlas of gene expression for ~9000 genes in the adult human cortex available in the AHBA dataset. Gene expression maps were obtained from the Neurosynth-Gene database (Supplementary Result 5). We performed partial least square (PLS) correlation analyses to identify the dominant gene expression patterns of functional variability (Fig. 3a). PLS correlation analyses ranked all ~9000 genes by their multivariate correlation with the variability distributions, resulting in one ranked gene list. These findings were corrected for spatial autocorrelation using the Moran Spectral Randomization (MSR)^42^ implemented in BrainSpace^43^, which generated spatially constrained null models for irregularly spaced data. Clear spatial correlation was evident between the first PLS (PLS1) component and the intersubject variability of WMFC (*r* = 0.30, *P*_moran_ = 0.0001; Fig. 3b), with high expressions (red areas) in the frontal networks and lower expressions (blue) in the cerebellum areas. In the gene list, expression patterns that were more strongly correlated with the corresponding intersubject variability map had large positive and negative PLS weights, and therefore, occupied more extreme ranks (Fig. 3c). Genes with strongly positive PLS weights showed positive spatial correlations between their expressions and variable WMFC organizations, and vice versa.

To investigate whether molecular biological signatures influenced the variable WMFC organization, we aligned the gene ontology (GO) biological processes and Kyoto Encyclopedia of Genes and Genomes (KEGG) pathways with the positive and negative PLS1 gene lists, respectively, using Metascape^44^. The top-ranked genes with positive PLS1 (PLS1+) weights were significantly enriched for several synapse-related terms and glutamatergic pathways (Fig. 3d), such as “chemical synaptic transmission”, “synapse organization”, “Glutamatergic synapse”, and “glutamate secretion”. However, the top ranked genes with negative PLS1 (PLS1–) weights were significantly enriched for glial cells-related pathways (Fig. 3e), including “ensheathment of neurons”, “regulation of gliogenesis”, and “axon development”. All these enrichment pathways were corrected with the false discovery ratio (FDR) with *P* < 0.05.

### Highly ranked genes expressed in specific cell types

The above findings indicated that the intrinsic transcriptomic organizations across the WM in healthy subjects were correlated with intersubject variabilities of WMFC. However, brain-wide transcriptional variations may reflect bulk samples of cells such as neurons, oligodendrocytes, astrocytes, microglia, endothelial cells, and oligodendrocyte precursors (OPCs)^45–47^. Therefore, we predicted that variable WMFC organization may be related to the expression patterns of variability-related genes across different cell types with varying spatial distributions. Because spatially comprehensive maps of cell type densities across the human brain were not available, we used previously defined cell-class gene sets to test whether the observed intersubject variabilities of WMFC were organized to broad cell-classes in the human WM^45^.

**Fig. 4:**
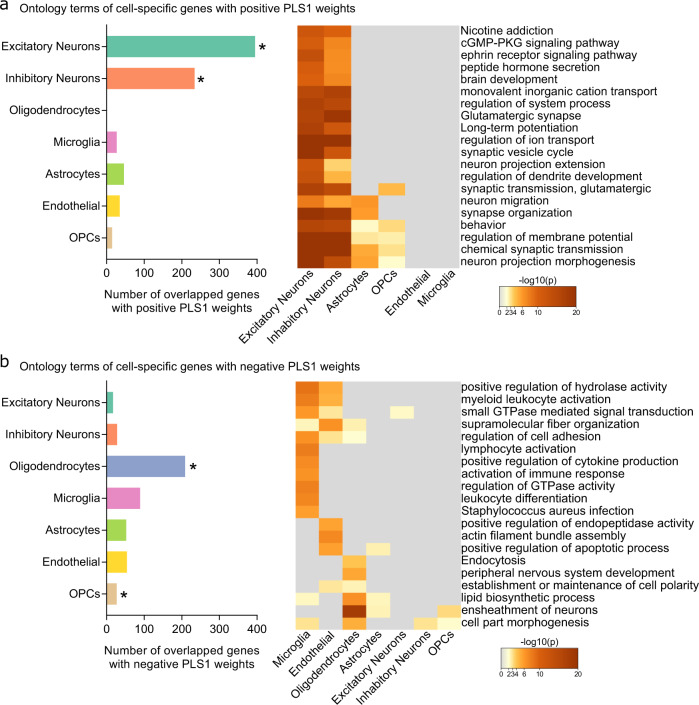
Cell type specificity of intersubject variability of white-matter functional connectivity (WMFC). Enrichment pathways of cell-specific highly-ranked genes with PLS1 + (**a**) and PLS1− (**b**) weights. Left panel is the number of highly-ranked genes in each cell type. Right panel is the enrichment analysis in each cell type. A key of heatmap indicates the statistically enriched terms in each cell type. *, *P*_perm_ < 0.05, false discovery rate-corrected. Source data provided as Source Data file.

We showed that the top ranked genes in the PLS1 + gene list were expressed primarily in neurons, both inhibitory (234 genes, *P*_perm_ = 0.0003, FDR-corrected) and excitatory (395 genes, *P*_perm_ = 0.0003, FDR-corrected) (Fig. 4a). Genes specific to oligodendrocytes (209 genes, *P*_perm_ = 0.0007, FDR-corrected) and OPCs (27 genes, *P*_perm_ = 0.0007, FDR-corrected) were also overrepresented in the top ranked PLS1– gene list (Fig. 4b). Confirming our approach, enrichment analyses using cell type specific genes in the PLS1 + gene list showed that intersubject variability of WMFC was significantly enriched for biological processes associated with neuronal cells. These were enriched for GO terms such as “Glutamatergic synapse”, “chemical synaptic transmission”, “synaptic vesicle cycle”, “synaptic transmission, glutamatergic”, and “synapse organization” (Fig. 4a). In a similar manner, enrichment pathways identified in cell type genes in the PLS1– gene list were usually concentrated in glial cells (Fig. 4b). Notably, these enrichments were more abundant in microglia, endothelial cells, and oligodendrocytes, suggesting a more pronounced physiological mechanism in these cells than that of the astrocytes and OPCs. Together, these results supported the validaty of our indirect approach in assigning gene expressions to unique cell types, and allowed us to detect specific cell types encoded in variable WMFC organizations.

### Genes enriched for brain diseases

**Fig. 5:**
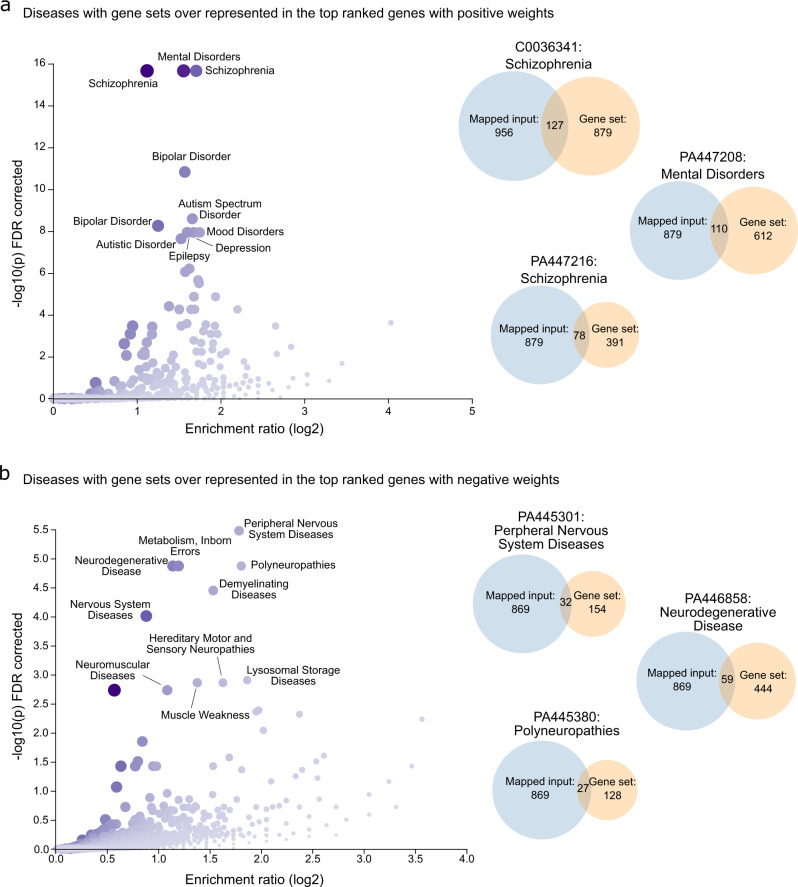
Highly-ranked genes enriched for brain diseases. Disease-association analysis in top ranked genes with PLS1 + (**a**) and PLS1− (**b**) weights. Volcano plots show disorders with gene sets that were overexpressed in top ranked genes of PLS1. Only the top 10 terms were annotated (*P*_FDR_ < 0.05). Venn diagrams show the top three overlapped diseases between the top gene set and genes sets for diseases. Source data provided as Source Data file.

To further characterize the importance of the putative variability of genes within the top ranked PLS1 + and PLS1− gene sets, we tested the hypothesis that genes related to brain WM disorders were enriched among genes associated with variable WMFC organizations. Using WebGestalt^48^, we combined Over-Representation Analysis (ORA) and Gene Set Enrichment Analysis (GSEA) of Disease Ontology (DO) terms based on DisGeNET, OMIM, and GLAD4U databases in the top-ranked PLS1 + and PLS1 − gene sets. We found a significant overlap between the top-ranked PLS1 + gene list and genes associated with psychiatric disorders, such as schizophrenia, bipolar disorder, autism spectrum disorder, and depression, all of which were FDR-corrected with *P* < 0.05 (Fig. 5a; Supplementary Result 6 and Supplementary Table 2). The results were consistent with previous studies that reported functional abnormalities of WM in major depressive disorders^49^, schizophrenia^50,51^, and autism spectrum disorder^52^. The PLS1− ranking genes were also enriched for neurological diseases, including peripheral nervous system diseases, neurodegenerative disease, polyneuropathies, demyelinating diseases, hereditary motor and sensory neuropathies, muscle weakness, and neuromuscular diseases (all FDR-corrected *P* < 0.05; Fig. 5b; Supplementary Table 3). These results were accompanied by enrichment pathways in cell type specific genes, indicating the different etiological mechanisms in PLS1 + and PLS1 − gene sets and the potential clinical applications of intersubject variability in WMFC. Considering multiple sclerosis as a typical disorder with abnormalities in WM, we also determined the relationships between WMFC intersubject variabilities and differentially expressed genes in multiple sclerosis^53^. We found that WMFC intersubject variability was correlated with dysregulated gene expression (*r* = –0.29, *P*_moran_ = 0.0002; Supplementary Result 7 and Supplementary Fig. 4), suggesting that multiple sclerosis involved differential impacts to areas along the WM distribution.

### Relationship between macroscale architecture and intersubject variability of WMFC

**Fig. 6:**
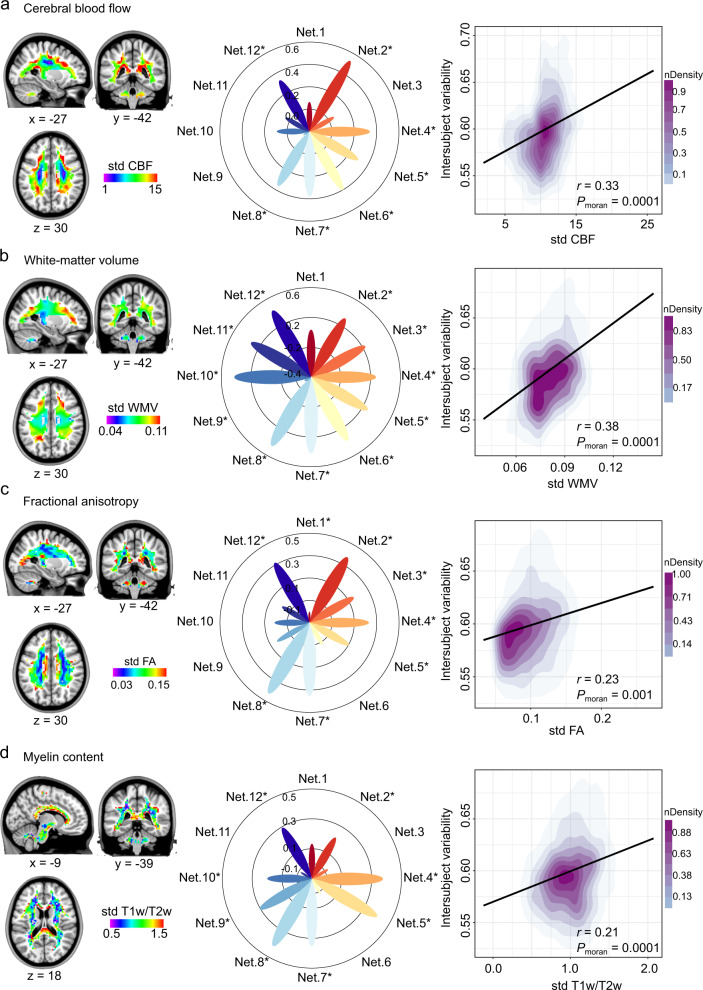
Intersubject variability of white-matter functional connectivity (WMFC) aligning with macroscopic imaging. **a** The distribution of intersubject variability of cerebral blood flow (CBF), and the relationship of intersubject variability between WMFC and CBF. **b** The distribution of intersubject variability of white-matter volume (WMV), and the relationship of intersubject variability between WMFC and WMV. **c** The distribution of intersubject variability of fractional anisotropy (FA), and the relationship of intersubject variability between WMFC and FA. **d** The distribution of intersubject variability of myelin content, and the relationship of intersubject variability between WMFC and myelin content. The left panel is the distribution of intersubject variability in each property. The middle panel is the association between functional variability in WM and variability of each neuroimaging index at the network level. Right panel shows the association between functional variability in WM and variability of each neuroimaging index at the whole brain WM level. The asterisk represents that WMFC intersubject variability correlated with the neuroimaging index variability within the WM functional network. Source data provided as Source Data file.

At the macroscale level, we sought to understand how variations of WMFC related to the hierarchical neurobiological organization of brain anatomical topography and the corticocortical connectivity pattern. Cerebral blood flow (CBF) regulation, with individual variability, is essential for brain function^8,54^. The frontoparietal control regions exhibit higher variability in CBF (Fig. 6a). Higher WMFC variability has been spatially associated with the higher variable of CBF (*r* = 0.33, *P*_moran_ = 0.0001, FDR-corrected). Specifically, the deep frontal WM network, temporal-orbitofrontal network, default mode network, dorsal attention network, superior longitudinal fasciculus system, visual superficial WM system and cerebellum system exhibited spatial correlations. These results indicated that the association regions with the greatest variabilities of WMFC involved a disproportionate degree of CBF across subjects.

Because almost half of the brain volume is WM, we computed the variability of WM volumes^55^, which was spatially correlated with the variability of WMFC in whole (*r* = 0.38, *P*_moran_ = 0.0001, FDR-corrected) and 11 of 12 networks, except for the frontoparietal control network (Fig. 6b).

Fractional anisotropy (FA) reflects fiber organization and axonal diameter in WM^56,57^. The structural variability of WM was consistent with the previous study reporting that superficial WM exhibited higher variability, whereas deep WM showed lower variability (Fig. 6c)^58^. Analysis of the association between structural variability of WM findings revealed a positive correlation in the default mode network, deep frontal WM network, temporal-orbitofrontal network, dorsal attention network, superior longitudinal fasciculus system, visual superficial WM system, and cerebellum system. When quantified on the whole brain surface, structural variability in WM showed a moderate but significant spatial correlation with the variability of WMFC (*r* = 0.23, *P*_moran_ = 0.001, FDR-corrected; Fig. 6c).

The T1w/T2w mapping has been proposed as a reliable method to measure the myelin content^59^ and appears related to the anatomical hierarchy in brain GM architecture^60^. In this study, visual and sensorimotor networks in the WM exhibited higher variable T1w/T2w ratios (Fig. 6d). We also assessed the relationships between variable WMFC organizations and variable T1w/T2w maps. We estimated the spatial correlations between variable T1w/T2w and WMFC and found that the deep frontal WM network, temporal-orbitofrontal network, default mode network, dorsal attention network, superior longitudinal fasciculus system, visual network, sensorimotor network, and cerebellum system exhibited correlations. Notably, a moderate positive correlation was also observed on a whole voxel level (*r* = 0.21, *P*_moran_ = 0.0001, FDR-corrected).

The consistent correlations of brain organizations between variabilities of the four neuroimaging indices and WMFC intersubject variability involved the deep frontal WM network, the temporal-orbitofrontal network/default mode network, the dorsal attention network, the superior longitudinal fasciculus system, and the visual network. The specificity of correlations in some brain WM functional organizations among the neuroimaging indices might indicate the different roles of brain properties in WMFC intersubject variability^8^.

We also assessed the short-range (i.e., local) and long-range (i.e., distant) corticocortical connectivities at each voxel tested for associations with intersubject variabilities of WMFC, as previously reported^10^. The percentage of local connectivity showed a moderate but significant correlation with the intersubject variability of WMFC (*r* = –0.24, *P*_moran_ = 0.02, FDR-corrected) across the entire voxels in WM (Supplementary Result 8 and Supplementary Fig. 5a), while the percentage of distant connectivity was not correlated with the intersubject variability of WMFC (*r* = –0.03, *P*_moran_ = 0.43; Supplementary Result 8 and Supplementary Fig. 5b).

### The role of intrinsic functional variations in behaviors

**Fig. 7:**
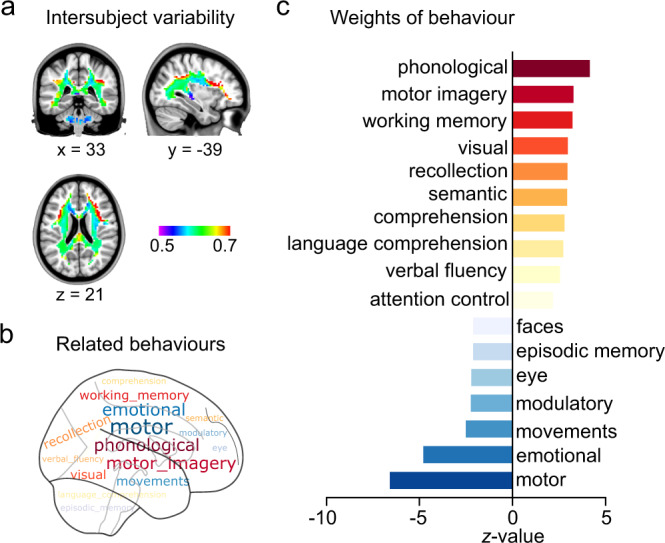
Functional variability in white-matter functional connectivity (WMFC) highly predictive of individual differences in cognitive traits. **a** Intersubject variability of WMFC. **b** Distribution of intersubject variability-related behaviors. **c** Weights of behavior. Source data provided as Source Data file.

To determine whether the variable WMFC organizations (Fig. 7a) were associated with behavioral performances, we performed a NeuroSynth meta-analyses. The relationships between cognition and interindividual variabilities is shown in Fig. 7b. Notably, we found a positive correlation between the functional variation and higher-order cognitive qualities (Fig. 7c), such as “phonological,” “motor imagery,” and “working memory”. Functional variability was negatively associated with lower-perceptual behavioral measures. Overall, these results supported the inference that interindividual WMFC variabilities had important implications for cognitive domains.

### Reproducibility of intersubject variabilities in WMFC

Because PLS is a model usually used for a predictive approach^61^, we validated the intersubject variability of WMFC based on the trained PLS predictive model. We used the variability data from four sessions, and estimated the intersubject variability in each session based on two runs. We then trained the PLS model on the first three sessions and tested it on the fourth session in a leave-one-out cross-validation scheme. We found that the intersubject variability based on the fourth session correlated with the predictive intersubject variability based on the first three sessions (*r* = 0.88, *P*_moran_ = 0.0001; Supplementary Result 9 and Supplementary Fig. 6).

In addition, we also verified the intersubject variability of WMFC using the four resting-state fMRI scans during 2 days, of the Human Connectome Project (HCP) 7 T dataset. We found that the intersubject variability of WMFC based on the HCP 7 T dataset showed a similar pattern with the intersubject variability across 12 WM functional networks in this work (*r* = 0.60, *P* = 0.04; Supplementary Fig. 7).

## Discussion

The current study characterized the endophenotyic intersubject variability of WMFC, revealing how brain function in WM was associated with phenotypic gene expression. These two biological parameters are complementary and mutually reinforcing. First, the spatial distribution was organized along a broadly hierarchical axis, anchored in unimodal regions and extending into heteromodal association regions. Second, the transcriptomic and cellular organizations of high and low variabilities of WMFC were distinct and specialized. Genes overexpressed in heteromodal association regions were expressed predominantly in neuronal cells, involved in synapse-related terms and glutamic pathways, and associated with common psychiatric disorders. However, genes overexpressed in unimodal regions were mostly expressed in glial cells and were associated with neurological diseases. Furthermore, the spatial distributions of functional variabilities were recapitulated in multi-modularity brain structural, functional, and behavioral organizations. Our findings provided a perspective on intersubject variability in brain function, which has potential implications for understanding brain evolution and development.

This study built on an earlier hypothesis that functional brain connectivity varied along with GM cortical gradients^8,10,11,38,62^. This variability has potential evolutionary significance, shaped by genetic and environmental factors^10^. The evolutionary trajectories are similar between brain GM and WM, which both originate from limbic specific and subcortical areas. They later include more associated networks for higher cognitive functions in WM^58,63^. Thus, it is reasonable to conclude that there are the similar spatially varying gradients of functional variabilities between GM and WM. However, the absence of an evolutionary WM expansion map has prevented us from directly verifying similar principles of functional variability in WM.

The transcriptome revealed variability-related biological processes, based on the complex pattern of functional topographies. Genes overexpressed in high and low variability areas were distinct and specialized, while genes overexpressed in high variability areas were enriched for synapse-related terms and glutamatergic pathways. Synapses in the central nervous system represent the classic mechanism through which neural cells communicate. Synaptic-style release of glutamate, the brain’s major excitatory neurotransmitter, occurs deep in the WM^64^. Here, glutamatergic pathways permit communication between axons and glial cells, enabling axon activity to couple with high fidelity to glial physiology^65–67^. The synaptic density changes with age^68^. Its overproduction is highest in prefrontal areas and lowest in primary sensory areas during development^69^. Thus, a gene overexpressed in synapse-related pathways may relate to highly functional variability in heteromodal association areas.

Genes with negatively PLS1 weights were mostly enriched for glia-related pathways. WM contains numerous glial cells. The biological process of regulation of gliogenesis results in the generation of glial cells^70^. In addition, ensheathment occurs in neurons in which glial cells envelop neuronal cell bodies and/or axons to form an insulating layer. This can take the form of myelinating or non-myelinating ensheathment. Myelin may serve as an inhibitor of brain plasticity^71^. This suggests that early sensory areas may require less plasticity, and therefore more myelin, whereas higher-order areas have less myelination, which might enable greater plasticity^72^. In the present study, we found that genes, overexpressed and underexpressed in areas with low and high variability in WMFC, respectively, were mostly enriched for glia-related biological processes. Together, this phenomenon may be caused by more myelination of neurons in low-perceptual areas, and more non-myelination of neurons in higher-cognitive areas.

The gene expression profiles characteristic of different cell-types arise because these cells have distinct sets of transcription regulators. We thus exploited how the cell type-specific manner affected gene regulation leading to intersubject variabilities in WMFC. When integrated across levels of cell types, these variability-related genes from bulk tissues were assigned to seven canonical cell classes^45^. Functional variability in WM was associated with genes expressed primarily in neurons (both inhibitory and excitatory) and oligodendrocytes, suggesting that intersubject variability may partially depend on synaptic transmission and myelinated processes^10,72^. The cellular organizations refined our transcriptomic analyses, suggesting the distinct contributions of neurons and glial cells, along with an inverse functional variability gradient. Critically, our analytic workflow identified cell classes and genes without relying on postmortem tissue from participants; thus, enabling investigators to make predictions regarding the human neuroscience or the biology of distinct human disorders, by using data from native human tissue^45^.

The clinical relevance of intersubject variability-related genes was further confirmed by over-representation analyses^73^. Genes enriched for synapse-related terms and glutamatergic pathways were mostly associated with various psychiatric disorders, such as depression, schizophrenia, and autism spectrum disorder. This was supported by a previous study linking gene transcription to synaptic activity processes to major psychiatric disorders^74^. Dysregulated synaptic development, properties, and plasticity have been hypothesized to result in altered neuronal function in various neuropsychiatric disorder^75,76^, suggesting that the disrupted neurobiological mechanisms are shared across common disorders^77^, and intersubject variability in WMFC may be a candidate intermediate phenotype for the association between these genes and some mental disorders.

Genes associated with microglia were significantly overlapped with neurological diseases. Microglia, the resident immune cells of the central nervous system, play an important role in maintaining tissue homeostasis and contribute to normal brain development^78^. Therefore, studying microglia provides unprecedented insight into mechanisms involved in neurological diseases^79,80^. Thus, microglial cells have the potential to act as diagnostic markers of disease onset or progression, and could contribute to the outcome of neurodegenerative diseases^81^. In this study, genes associated with microglia were overexpressed in low variability areas, indicating that these brain areas may contribute to neurological diseases. Our results showed that variability-related gene data can relate to knowledge about transcriptome-neuroimaging, and may be clinically translatable.

Leveraging macroscale neuroimaging, we found that intersubject variability in WMFC recapitulated the variability of structural (i.e., WMV, FA, and T1w/T2w) and functional (i.e., CBF) organizations. From an evolutionary point of view, the heteromodal association areas that have undergone dramatic cortical expansion showed higher metabolic demands, more structural variability, and greatly reduced myelination, when compared with unimodal areas^8,58^. It is striking how well the CBF and structural variability maps matched the distribution of intersubject variability in WMFC, as revealed in this study. These findings suggested that the different scales of human brain organization are likely not independent from each other during brain development^29^.

The reasons for differences in the molecular hierarchies observed in the variable functional organizations between WM and GM are not completely understood. Using transcriptomic analysis and cellular specificity of the intersubject variability in WMFC, we found specific and common enrichment pathways of intersubject variability-related genes between WMFC and gray-matter FC (Supplementary Result 10 and Supplementary Fig. 8). Although WM contains a significantly lower neuron-to-glia ratio than GM^82^, synapse-related terms are commonly enriched for functional variabilities both in WM and GM, indicating the important roles of whole brain functional variability for psychiatric disorders. In addition, intersubject variabilities of WMFC organization exhibited specific enrichment pathways in glial cell-related terms, such as ensheathment of neurons and regulation of gliogenesis, highlighting the importance of functional variabilities in WM, as compared with GM, for neurological diseases. Understanding the nuances of shared and specific pathways of variabilities between WM and GM will increase our understanding of human neuroscience and may provide avenues of treatment for several diseases.

Several methodological considerations are noteworthy. First, we collected longitudinal, multi-session resting-state BOLD-fMRI data to ensure stable and reliable intersubject variability in WMFC. In addition, considering the spatial and temporal limitations of BOLD-fMRI data based on a 3 T machine, we further used four sessions of the HCP 7 T dataset to verify the intersubject variability of WMFC. Second, we used several complementary analysis strategies to obtain clean BOLD-fMRI signals from the WM, by strictly controlling the boundary between WM and GM, by separating WM and GM functional signal in preprocessing, and by identifying participants’ voxels only in WM to create a WM mask^17,19–21,49^. Furthermore, from the architecture of brain venous systems, the possibility that deoxygenated blood was drained from cortical GM to deep WM was significantly small^83^. Moreover, there are two venous systems in normal neuroanatomy; one is the superficial venous system, which drains deoxygenated blood in the GM cortex and superficial WM into pial veins. The other is a deep system draining deoxygenated blood in deep WM into subependymal veins^83,84^. Furthermore, the brain venous architectures are spatially non-overlapping. Deoxygenated blood drainage from GM cortex to deep venous system through WM does exist, but the probability of draining is less than 3%^83,84^. Collectively, based on brain venous system architecture, these approaches ensured that BOLD-fMRI signals analyzed in the present study were from WM.

Several limitations in this study need to be considered. First, all subjects were college students, so a larger sample from a community-based population of varying ages is needed to generalize the current findings. Second, the AHBA gene data were measured postmortem in six subjects, which limited examinations of transcriptome-neuroimaging associations across groups and possibly placed individual effects out of scope. In addition, the AHBA gene data only included the right hemisphere for two subjects, and contained relatively small probes in WM, limiting our investigation between genes and functional variability in whole brain WM. Finally, the PLS analysis may result in inaccurate associations when the number of samples per feature is relatively small. In future studies, we will first perform data reduction to solve this problem^85^ by selecting genes based on prior hypotheses.

In this study, we have proposed a landscape of the transcriptomic decoding and cellular specificity of intersubject variability in WMFC—high-to-low functional-varying gradients that were associated with gene expressions, associated with neurons to glial cells. Notably, synapse-related and glia-related genes were overexpressed in association and low-order perceptual networks, respectively, which were specifically related to several neuropsychiatric illnesses and neurological diseases. In addition, the shared and specific biological processes between WM and GM highlighted the complementary and reverberating functional roles. These findings emphasized the clinically translational relevance for incorporating the specific intersubject variabilities in WM and GM to determine effective therapeutic targets.

## Methods

### Study design and participants

This longitudinal study was approved by the Local Medical Ethics Committee of the University of Electronic Science and Technology of China (UESTC), China. Written informed consent was obtained from all subjects prior to scanning. Forty-five healthy subjects [age (mean ± SD): 23.67 ± 1.65 years, 20 females, all right-handed] were recruited from and the data were collected at the UESTC. All subjects had no history of neurological or psychiatric conditions, and no gross abnormalities on brain MRI.

Each subject underwent four MRI scanning sessions within 6 months (~14, 30, and 180 days from enrollment). All subjects performed two resting-state fMRI runs per session to estimate the WMFC. After quality control, 43 subjects who had two good runs (mean FD < 0.2 mm) in each session and two subjects who had one good run in one of four sessions were included for subsequent analyses.

### Data acquisition

Multimodal neuroimaging battery (resting-state functional, structural, and diffusion data) were acquired on a 3.0 Tesla MRI scanner (GE Discovery 750 MRI) at the UESTC. During the resting-state functional scanning, subjects were instructed not to think of anything particular, to keep their eyes open, and to stay in a fixed position.

*Resting-state BOLD-fMRI scan*: To characterize intersubject variability of WMFC, each subject first underwent two resting-state BOLD-fMRI runs (30 min 10 s with 905 volumes per run) using an echo-planar imaging sequence: repetition time (TR)/echo time (TE) = 2000 ms/30 ms, flip angle = 90°, field of view (FOV) = 240 × 240 mm^2^, in-plane matrix = 64 × 64, slice thickness = 3.2 mm, no interslice gap, and 43 transverse slices. The identical protocol was used for each session.

*Resting-state arterial spin labeling (ASL) scan*: To associate the spatial pattern of intersubject variability of WMFC, 34 out of 45 subjects underwent one resting-state ASL run during the last session using a 3D pulsed continuous ASL technique. Interleaved 30 pairs control and label images were acquired (7.5 min with 50 of control and label images). We used the following settings: TR/TE = 4632 ms/10.536 ms, flip angle = 111°, label duration = 1450 ms, post-labeling delay = 1525 ms, FOV = 240 × 240 mm^2^, in-plane matrix = 128 × 128, slice thickness = 4 mm, no interslice gap, slices = 36, acquisition time = 269 s.

Subsequently, structural, myelin, and diffusion data were obtained. All subjects, during each session, underwent high-resolution T1-weighted (T1w) structural scans using a 3D Fast spoiled gradient-recalled echo sequence in the sagittal orientation (TR/TE = 5.952 ms/1.96 ms, flip angle = 12°, FOV = 256 × 256 mm^2^, in-plane matrix = 256 × 256, slice thickness = 1 mm, and 154 slices) and diffusion-weighted images (DWI) using a spin echo-based echo-planar imaging sequence (TR/TE = 8500/64.7 ms, flip angle = 90°, FOV = 256 × 256 mm^2^, in-plane matrix = 128 × 128, slice thickness = 2 mm, slices = 78, four volumes without diffusion weighting *b* = 0 s/mm^2^, and 60 non-collinear directions *b* = 1000 s/mm^2^). In addition, 44 out of 45 subjects also underwent T2-weighted structural scans during the last session using a 3D Cube T2 sequence in the sagittal orientation with TR/TE = 2500 ms/60.97 ms, flip angle = 90°, FOV = 256 × 256 mm^2^, in-plane matrix = 256 × 256, slice thickness = 1 mm, and 256 slices.

### Data preprocessing

Both BOLD-fMRI and ASL images were preprocessed using the statistical parametric mapping toolbox (SPM12; https://www.fil.ion.ucl.ac.uk/spm/software/spm12/), as outlined below. The resting-state BOLD-fMRI data were preprocessed according to our previously described WM functional procedures^19,20,49^ based on DPARSF (v4.3, http://rfmri.org/DPARSF). For each run, slice-timing correction and realignment were applied to the 900 functional volumes after excluding the first five volumes (10 s). For each subject, the mean FD was measured to control data quality. Each subject’s structural image was co-registered to the preprocessed functional images, and was then segmented into GM, WM, and cerebrospinal fluid (CSF) using DARTEL^86^. The mean signals from CSF, 24 head motion parameters (Friston 24-parameter model) were regressed out by multiple linear regression analysis. To avoid elimination of important neural signals, we did not regress out WM and brain global signals, as previous studies have suggested^17,19–21,49^.

To minimize mixing signal (and noise) components from the WM and GM due to partial volume effect, subsequent processing of the functional images was performed separately for WM and GM, in accordance with our previous studies^19,20,49^. First, each individual mask was generated using a 60% threshold on the probability map of WM (i.e., produced by structural segmentation). Second, functional images were spatially divided into WM functional images using the dot product between functional images and the individual WM mask. Third, the WM functional images were spatially normalized to a standard space (Montreal Neurologic Institute, MNI) by structural segmentation and were resampled to 3 × 3 × 3 mm^3^. Then, only voxels identified as WM across all of the participants were used to create the group-level WM mask. To minimize the impact of deep brain structures, the probability (25% threshold) Harvard-Oxford Atlas was used to remove subcortical structures (i.e., the bilateral thalamus, putamen, caudate, pallidum, and accumbens) from the group-level WM mask as previous studies suggested^17,19,20,49^. Subsequently, functional data preprocessing included smoothing with 6 mm full-width half-maximum (FWHM) isotropic Gaussian kernel, and a band-pass filtering (0.01–0.10 Hz).

For ASL images, the absolute quantified rCBF maps were first obtained by subtracting the label from control images using Functool (v 12.2.01) embedded in the GE MR-750 scanner system. Then, the z-scored rCBF maps were performed by SPM12 according to the following steps: firstly, T1w images were coregistered to their own rCBF maps, and then segmented into GM, WM, and CSF. Second, the WM maps were nonlinear co-registered to the T1 template in MNI space. Finally, each rCBF map was normalized into the standard MNI space with 3 mm isotropic voxels based on the transform parameters that were estimated during nonlinearly co-registration. he normalized rCBF maps were spatially smoothed by a 6 mm FWHM Gaussian kernel.

The DWI data were preprocessed on volumetric space using FSL (v6.0, https://fsl.fmrib.ox.ac.uk/fsl/fslwiki). For each subject’s native space, the DWI images were corrected for the eddy-current-induced distortions and head movements, and the diffusion tensor model was estimated using the linear least-squares fitting method, resulting in the fractional anisotropy (FA) map. Individual T1-weighted images were co-registered to the images in the DTI native space. Then, the co-registered T1-weighted image was mapped to the T1 image in MNI space by applying an affined transformation to obtain the transformation matrix^87^. Finally, FA maps were normalized into the standard MNI space and resampled with 3-mm isotropic voxels based on the inverse transformation matrix.

To obtain myelin enhanced contrast images, the T1w/T2w ratio was calculated for the same subject using MRTOOL (https://www.nitrc.org/projects/mrtool/)^56^. To standardize the T1/T2w image, we created two subject-specific masks by warping predefined masks in the MNI standard space to individual space. One mask contained relatively low values on the T1w image and high values on the T2w image, and the other mask had reversed characteristics. We implemented this specification by selecting two masks covering the eyeballs and temporal muscles, respectively^56^. These masks were defined directly in the MNI space by segmenting and thresholding the ICBM152 template images^56^. In parallel, the T2w image was co-registered to the T1w image. Then, the T1w and T2w images were jointly subjected to bias correction to ensure that the sensitivity profile was spatially equalized. After calibrating T1w and T2w images, their ratio was calculated to produce the calibrated T1w/T2w image as a myelin enhanced contrast image.

### Intersubject variability of WMFC

After preprocessing, BOLD-fMRI data were concatenated into a single run (900 × 2 volumes) for each session^10^ if the subject had two good runs. After obtaining an individual WM mask for each subject, these individual masks were averaged across participants to obtain the percentage of participants in which it was classified as WM for each voxel. Voxels identified in all sessions and all participants as WM were considered as a group-level WM mask, resulting in 7151 voxels^19,20,49^.

Intersubject variability of WMFC was estimated in line with previous studies on functional variability in GM^10,11,38^. To obtain individual WMFC maps, we used each of the 7151 voxels as the seed, and computed the FC between the seed and the remaining voxels, resulting in 7,151 maps for each subject and each session. This individual correlation map based on each seed voxel was vectored as *WMFC*_*v*_(s, t), where *v* = 1, 2, …, 7,151, the values represented the FC between the seed *v* and the remaining voxels; *s* $(s\in 1,2,\ldots ,45)$ denoted the number of subjects; and *t* $(t\in 1,2,\ldots ,4)$ denoted the number of sessions.

For each session, 45 maps derived from 45 subjects were obtained. For a given seed voxel, *v*, the spatial similarity between the 45 maps derived from 45 subjects was quantified by averaging Pearson’s correlation coefficients between any two WMFC vectors (total, ${C}_{45}^{2}=990$ combinations):

$${R}_{v}(t)=E[corr(WMF{C}_{v}({s}_{p},t),WMF{C}_{v}({s}_{q},t))],$$

where, *p* and *q* indicate two different subjects, $p,q=1,2,\ldots ,45;p\,\ne\, q$. This resulted in an intersubject similarity map.

To obtain the intersubject variability of WMFC, we first adjusted spatial similarities between the 45 maps for each session:

$${R}_{v}^{{\prime} }(t)=1-{R}_{v}(t)$$

Finally, four original (unadjusted) intersubject variability maps were obtained.

The intra-subject variability was measured based on repeated multi-sessions (*t* = 4) of a subject. For each subject, four WMFC vectors were obtained from four sessions. Accordingly, the spatial similarities of intra-subjects were estimated using any two WMFC vectors (total, ${C}_{4}^{2}=6$ combinations). Considering the values of WMFC maps ranging from ‒1 to 1, we inverted the similarity map by subtracting from one (i.e., 1 – *R*_spatial similarity_) as the intra-subject variability of each subject:

$${N}_{v}(s)=1-E[corr(WMF{C}_{v}(s,{t}_{m}),WMF{C}_{v}(s,{t}_{n}))],$$

where $$m,n=1,2,\ldots ,4;m\,\ne\, n$$.

The intra-subject variability map was then averaged across 45 subjects and assigned to the seed voxel, *v*:

$${N}_{v}=E[{N}_{v}(s)]$$

“Technical noise” of the voxel *v* was reflected by the *t*SNR of the BOLD-fMRI signal:

$$tSN{R}_{v}=\frac{ < S{ > }_{t}}{{\sigma }_{t}}$$

Where <*S*>_*t*_ is the average BOLD signal across time, and *σ*_*t*_ is the corresponding temporal standard-deviation map. The technical noise was the inverse *t*SNR (i.e., 1/ *t*SNR).

To minimize the effects of confounding factors on intersubject variability, the intra-subject variability and “technical noise” were regressed out from original (unadjusted) ${R}_{v}^{{\prime} }(t)$ using a general linear model (GLM) for adjusting intersubject variability:

$${V}_{v}(t)={R}_{v}^{{{\prime}}}(t)-{\beta }_{1}\times {N}_{v}-{\beta }_{2}\times Noise-c,$$

where ${\beta }_{1},{\beta }_{2},and\,c$ are parameters determined via a GLM. Finally, resulting adjusted maps derived from each session *t* were averaged for intersubject variabilities of the WMFC map (Fig. 2).

### Functional organization of intersubject variability

To quantify the distribution of intersubject variability within each of functional network in WM, we used the previous parcellations based on healthy controls to identity large-scale networks as reported by Peer et al.^17^. Considering non-zero voxels of the WM mask in this study, we first overlapped the WM mask with the previous functional parcellations in WM. We then assigned each voxel to one of the functional networks based on the MNI coordinate, and rearranged the network label. According to the previous study^17^, the function of each WM network was described as follows: Network 1 was the frontoparietal control network and default mode network (n = 160 voxels); Network 2 was the deep frontal WM network (n = 1293 voxels); Network 3 was the inferior longitudinal fasciculus system (n = 777 voxels); Network 4 was the temporal-orbitofrontal network and default mode network (n = 112 voxels); Network 5 was the dorsal attention network (n = 717 voxels); Network 6 was the forceps minor system (n = 233 voxels); Network 7 was the superior longitudinal fasciculus system (n = 2135 voxels); Network 8 was the visual superficial WM system (n = 40 voxels); Network 9 was the sensorimotor superficial WM system (n = 90 voxels); Network 10 was the sensorimotor and ventral attention system (n = 475 voxels); and Network 11 (n = 224 voxels) and Network 12 (n = 873 voxels) were cerebellum systems. Finally, the intersubject variability of voxels within each WM functional network was averaged as the intersubject variability of the WM functional networks.

### Investigating the gene expression pattern associated with the intersubject variabilities of WMFC

The AHBA dataset bridged the gap between macroscale intersubject variability of WMFC and microscale gene expression^46^. The AHBA microarray-based gene expression provided high resolution coverage of nearly the entire brain, with 3702 spatially distinct tissue samples taken from six neurotypical postmortem brains. The AHBA microarray data were processed including verifying probe-to-gene annotations, filtering of probes, and probe selection following the protocol of Arnatkevic *et al*.^88^. After preprocessing, 10,027 genes lists were used to select gene expression maps from Neurosynth-Gene (https://www.neurosynth.org/genes/). These gene expression maps covered the whole brain and co-registered the locations of all microarray samples with the MNI stereotactic space. Consequently, the overlapped 9922 gene list were used for further analyses. In addition, the number of overlapped voxels between gene expression and intersubject variability maps was 3415, resulting in a matrix (3415 voxels × 9922 genes) of the brain-wide gene expression for the WM.

PLS correlation^89^ was used to determine the relationship between the intersubject variability of WMFC and transcriptional activity for all 9,922 genes. Gene expression data were used as predictor variables of intersubject variability in the PLS regression. PLS1 was the linear combination of gene expression values, which was most strongly correlated with functional variability in WM. Findings were corrected for spatial autocorrelation with Moran Spectral Randomization (MSR)^42^ implemented in BrainSpace^43^, which generated spatially constrained null models for irregularly spaced data. Bootstrapping was used to estimate the error of each gene PLS1 weight, and the ratio of the weight of each gene to its bootstrap standard error was used to calculate the *Z* scores and to rank the genes according to their contributions to PLS1^90^. The set of genes with a FDR of 5%, either positive (PLS1 + ), or negative (PLS1−), was identified for the intersubject variability gene list.

### Gene annotation analyses: functional enrichment

Functional enrichment for GO gene sets and the KEGG pathway search were used to include over 40 independent knowledgebases within Metascape^44^. The lists of PLS1 + and PLS1− genes were separately analyzed using Metascape (https://metascape.org/gp/index.html#/main/step1). Based on the well-adopted hypergeometric test, Metascape identified all ontology terms that contained a statistically greater number of genes in common with an input list than expected by chance. Briefly, pairwise similarities between any two enriched terms were computed based on a *Kappa*-test score^91^. The similarity matrix was then hierarchically clustered to trim the constructed tree into separate clusters. The resulting enrichment pathways were thresholded for significance at 5%, corrected by the FDR.

### Genes expressed in specific cell types

To further refine our analysis and considering cellular diversity in the brain, we took an indirect approach to assign PLS1 + and PLS1− genes to seven canonical cell classes. To obtain gene sets from each cell type, we compiled data from five different single-cell studies using postmortem cortical samples in human postnatal subjects. This approach avoided any bias based on acquisition methodology, analysis or thresholding, leading to the initial inclusion of 58 cell classes, which were provided by Seidlitz et al.^45^. Many of those cell classes were overlapping based on nomenclature and/or constituent genes. To avoid any bias based on acquisition, analysis, or thresholding, five different single-cell studies were included in this study^92–96^. We further organized cell types into seven canonical classes following Seidlitz et al.^45^. These cell types included microglia, endothelial cells, oligodendrocyte precursors, oligodendrocytes, astrocytes, and excitatory and inhibitory neurons. Only one study included the annotation of the pericyte type, thus this gene set was excluded. To assign intersubject variability-related genes obtained by PLS analysis to cell types, we overlapped the gene set of each cell type with the PLS1 + or PLS1− rank gene list. We resampled the genes involved in cell types 5000 times to test the null hypothesis that the PLS1 + or PLS1− gene list was randomly assigned to different cell types. The *p*_perm_ scores were obtained by the occupied null models (<5th, or >95th centile) and corrected by FDR.

### Genes enriched for brain diseases

Brain disease-related enrichment for intersubject variability-related genes was determined using WebGestalt^48^ (http://www.webgestalt.org/) to identify disease-association terms based on DisGeNET, OMIM, and GLAD4U databases. The gene lists of PLS1 + and PLS1− were curated for brain disease gene associations. The top 10 terms were listed according to the ranked *P* < 0.05, FDR-corrected.

### Relationship between macroscale functional and anatomical variability and WMFC variability

The rCBF has received much attention in brain function-related studies due to its important role in maintaining normal brain function and its close relationships with brain metabolism and connectivity^8^. Canonical structural variability indices in WM were white matter volume (WMV) and FA variabilities^58^. The myelin content representing T1w/T2w was an important component in WM. Thus, intersubject variability in these functional and anatomical indices was estimated as voxel-wise using SD across subjects^5,55,97^. The Spearman’s correlation coefficient was calculated between the intersubject variability map in WMFC and these macroscale indices for across-voxel in the WM.

### Behavior decoding of the intersubject variabilities of WMFC

Functional activation probability maps were obtained for multiple cognitive terms using the Neurosynth meta-analytic database using volumetric “association test” maps^98^ (https://www.neurosynth.org). Then, each cognitive map was overlapped with the intersubject variability of the WMFC map to obtain the non-zero voxels between the two maps. Pearson’s correlation analysis was used to measure the similarity between the cognitive map and intersubject variability of the WMFC map. Terms were selected in this study if their thresholds of *z*-statistic values were above 2.1 (corresponding Pearson’s correlation coefficient: *P* < 0.05), and if they were not “noise” terms, which did not capture any coherent cognitive function^99^.

### Statistics and reproducibility

Testing for linear associations between the intersubject variability of WMFC and other brain phenotypes may lead to biased testing statistics due to spatial autocorrelation of MRI data. Thus, we generated random datasets, with equivalent spatial autocorrelations as the response variables using MSR and the singleton procedure^42^ implemented in BrainSpace^43^. The MSR methods generated spatially constrained null models for irregularly spaced data. All linear models were fitted for the original data as well as 10,000 corresponding simulated datasets. *P*_moran_-values were obtained by the occupied null models (<5th, or >95th centile) and corrected by FDR.

Reproducible analyses were used to validate the intersubject variability in WMFC. We first built trained PLS predictive model based on the variability data from four sessions, where each session included two runs. The first three session were trained on the PLS model and the fourth session were uses as test data in a leave-one-out cross-validation scheme. To test the predictive power, correlation analysis was used between the predictive and observed intersubject variability. In addition, HCP dataset was used te test the robustness of the intersubject variability of WMFC.

### Reporting summary

Further information on research design is available in the Nature Research Reporting Summary linked to this article.

## Supplementary information

Supplementary Information

Description of Additional Supplementary Files

Supplementary Data 1

Reporting Summary

Editorial Policy Checklist

## Data Availability

Human gene expression maps that support the findings of this study are available Neurosynth-Gene database (https://www.neurosynth.org/genes/) based on the Allen Brain Atlas (https://human.brain-map.org/static/download). Compiled cell-specific gene set list from all available large-scale single-cell studies of the adult human cortex can be obtained from the raw Seidlitz et al.^45^ dataset (https://static-content.springer.com/esm/art%3A10.1038%2Fs41467-020-17051-5/MediaObjects/41467_2020_17051_MOESM8_ESM.xlsx). Disease-association terms were obtained from WebGestalt website (http://www.webgestalt.org/). The 7 T Human connectome dataset is available at https://db.humanconnectome.org/. The neuroimaging data that support the findings of this study are available from the corresponding author (W.L.) upon reasonable request. The source data underlying Figs. 2, 3, 4, 5, 6, and 7 are provided as Supplementary Data 1.

## Code avaliability

The code for gene expression analysis can be found at https://github.com/BMHLab/AHBAprocessing. Gene enrichments were analyzed at https://metascape.org/gp/index.html#/main/step1. The code for spatial permutation testing was implemented in BrainSpace (https://www.brainspace.com/). The code for preprocessing of WM functional data can be found at https://github.com/weiliao81/WMFPrep.
