# Supplementary material for: Transcriptomic and macroscopic architectures of intersubject functional variability in human brain white-matter
Source: Commun Biol. 2021 Dec 20;4:1417. doi: 10.1038/s42003-021-02952-y (PMC8688465; doi:10.1038/s42003-021-02952-y)
Supplement: Supplementary file 1 — Supplementary Information [file 42003_2021_2952_MOESM1_ESM.pdf]

Supplementary material for

Transcriptomic and macroscopic architectures of intersubject functional variability in human brain white-matter

Jiao Li[1,2,3], Guo-Rong Wu[4], Bing Li[1,3], Feiyang Fan[1,3], Xiaopeng Zhao[1,3], Yao Meng[1,3], Peng Zhong[1,3], Siqi Yang[1,3], Bharat B. Biswal[1,3,5], Huafu Chen[1,2,3,✉], Wei Liao[1,3,✉]

[1] The Clinical Hospital of Chengdu Brain Science Institute, MOE Key Laboratory for Neuroinformation, University of Electronic Science and Technology of China, Chengdu 611731, P.R. China.

[2] The Center of Psychosomatic Medicine, Sichuan Provincial Center for Mental Health, Sichuan Provincial People's Hospital, University of Electronic Science and Technology of China, Chengdu 611731, P.R. China.

[3] School of Life Science and Technology, Center for Information in BioMedicine, University of Electronic Science and Technology of China, Chengdu 611731, P.R. China.

[4] Key Laboratory of Cognition and Personality, Faculty of Psychology, Southwest University, Chongqing 400715, P.R. China.

[5] Department of Biomedical Engineering, New Jersey Institute of Technology, Newark, NJ 07103, USA.

✉ Corresponding authors:

Huafu Chen (chenhf@uestc.edu.cn), The Center of Psychosomatic Medicine, Sichuan Provincial Center for Mental Health, Sichuan Provincial People's Hospital, University of Electronic Science and Technology of China, Chengdu 611731, P.R. China.

Wei Liao (weiliao.wl@gmail.com), The Clinical Hospital of Chengdu Brain Science Institute, School of Life Science and Technology, University of Electronic Science and Technology of China, Chengdu 611731, P.R. China.

Supplementary Results

1. Data description

The current work used a hybrid design (i.e., within + between design) in which scans repeated one or more times on same day, as well as across one or more sessions [1]. Each subject participated in four sessions over about six months. The mean interval between session 1 and 2, between session 2 and 3, and between session 3 and 4 were 14.29 days, 18.87 days, and 150.64 days, respectively. Each session included two runs (interval = 4 minutes). The duration of each run was 30 min 10 s, including 905 volumes. The data description is shown in Supplementary Figure 1.

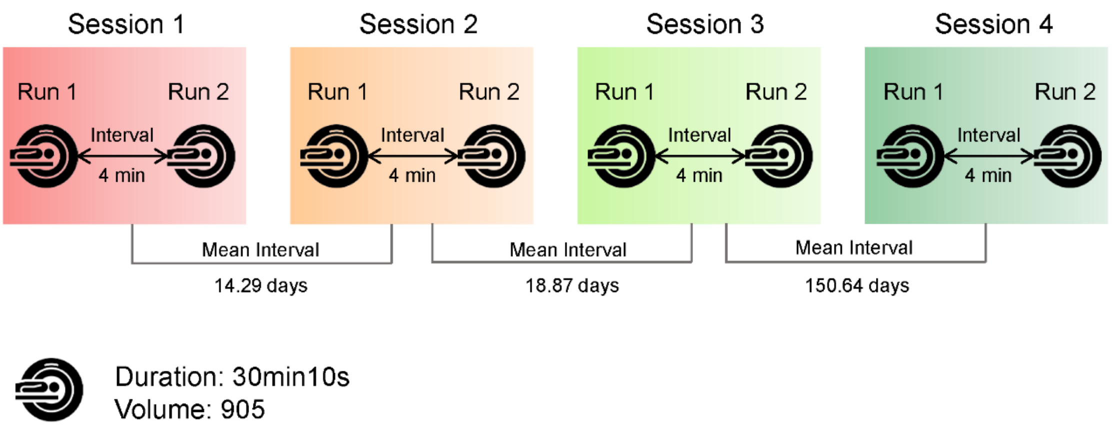

Supplementary Figure 1. Data description for the current work.

2. Image quality control

To measure the quality of the fMRI data, we calculated a series of common quality metrics using MRIQC [2, 3] (https://github.com/nipreps/mriqc), including spatial information [Entropy Focus Criterion (EFC), Foreground to Background Energy Ratio (FBER), Signal-to-noise ratio (SNR)], and temporal information [temporal SNR (tSNR), and Mean Fractional Displacement (mean FD)] (Supplementary Figure 2). Information about these image quality metrics can be found in MRIQC's documentation.

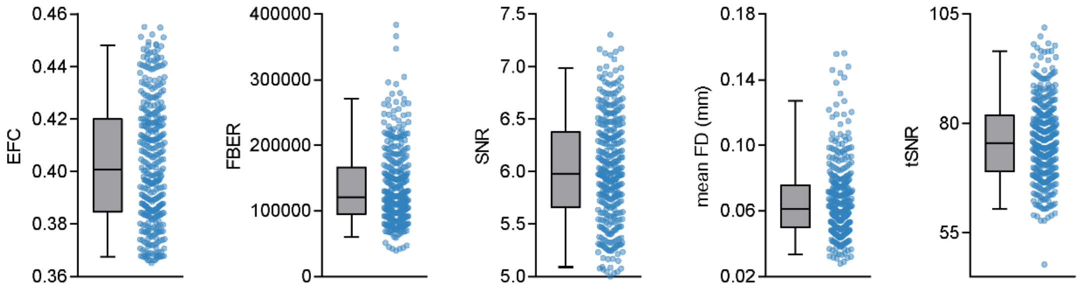

Supplementary Figure 2. Distribution of image quality metrics of resting-state fMRI data.

3. Intersubject variability not accounting for intrasubject variability

Before accounting for intrasubject variability, we found that the association networks, including temporal-orbitofrontal network, default mode network, frontoparietal network, and dorsal attention network, exhibited a relative high intersubject variability (Supplementary Figure 3a). Both intersubject variability and intrasubject variability were nonuniformly distributed across WM functional networks. The intrasubject variability was estimated using the four maps derived from four scanning sessions of each subject. Consistent with the previous studies focused on GMFC [4, 5], most association networks exhibited lower intrasubject variability than sensory-motor and visual networks (Supplementary Figure 3b). In addition, intersubject variability was much higher than intrasubject variability, as previously reported [4].

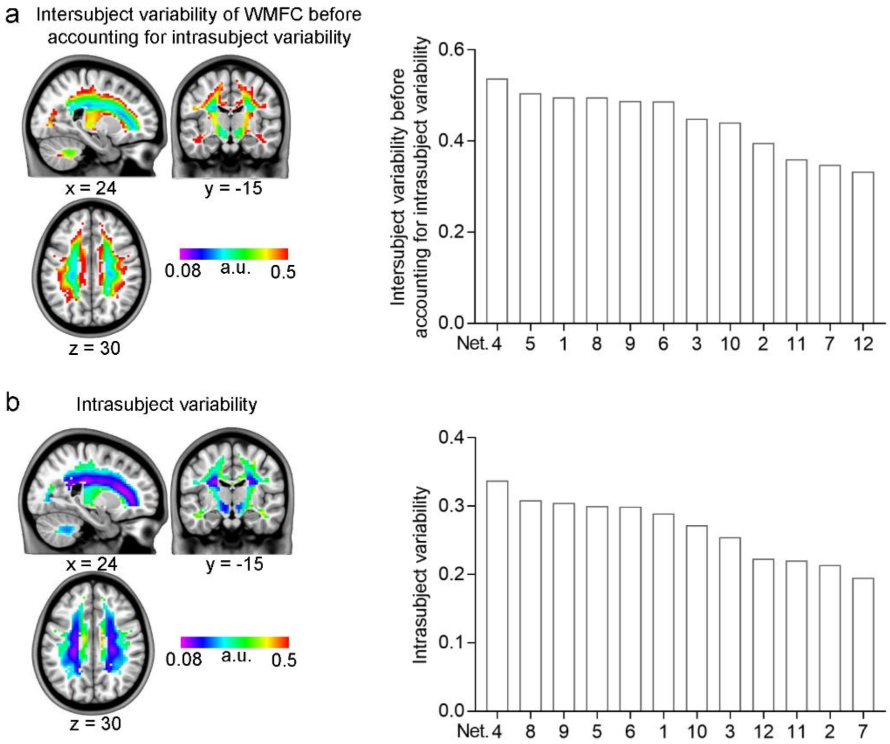

Supplementary Figure 3. Visual and sensory-motor networks exhibited lower intersubject, but higher intrasubject variability of WMFC than most association networks. a Intersubject variability before accounting for intrasubject variability for different WM functional networks. **b** Distribution of intrasubject variability for different WM functional networks. The network labels are identical to that definded in Figure 2.

4. Gene expression of AHBA atlas

We used the Allen Human Brain Atlas (AHBA) dataset (http://human.brain-map.org)—a whole-genome, whole-brain transcriptomic dataset to obtain brain gene expression [6]. The AHBA dataset includes six neurotypical adult brains, three Caucasian, two African-American, and one Hispanic. Their ages range from 24 to 57 years (age = 42.5 ± 13.38 years; male/female = 5/1). The detailed information is shown in Supplementary Table 1.

Supplementary Table 1. Demographics of six adult donors in AHBA dataset

Donor	Number of samples	Age	Sex	Ethnicity	Post-mortem interval [a]
H0351.2001[b]	946	24	Male	African American	23h
H0351.2002[b]	893	39	Male	African American	10h
H0351.1009	363	57	Male	Caucasian	25.5h
H0351.1012	529	31	Male	Caucasian	17.5h
H0351.1015	470	49	Female	Hispanic	30h
H0351.1016	501	55	male	Caucasian	18h

Note:

[a] Post-mortem interval is defined as the time period from the time of death to the time the tissue is frozen.

[b] These donors have tissue samples collected across left and right hemispheres, while the other donors have tissue samples only in left hemisphere.

5. Estimation of gene expression in brain white matter

Firstly, followed by Arnatkevic *et al*. [7], we focused on expression profiles of 10,027 genes surpassing quality-control criteria including: i) verifying probe-to-gene annotations. Probe-to-gene annotations were provided by Arnatkevic *et al*. [7] using Re-annotator toolkit [8]. After the reannotation, a final set of 45,812 probes were uniquely annotated to gen and could be related to an Entrez ID. Sequent analysis used the re-annotated set of 45,821 probes, corresponding to 20,232 unique genes; ii) filtering of probes that do not exceed background noise. To improve the validity of microarray expression measures, we used an intensity-based filtering strategy [6, 9, 10]. Here, probes that do not exceed the background in at least 50% of all samples across all subjects were excluded; and iii) probe selection. Generally, multiple probes can be used to measure the expression level of a single gene at different exons, in these cases, the probe with the highest correlation to RNA-seq data is selected [11]. The resulting of the gene number was 10,027.

To explore the gene expression in brain white matter (WM), we then downloaded 19,695 gene expression maps from Neurosynth-Gene (https://www.neurosynth.org/genes/). The subsequent transcriptomic analysis used all common genes across Neurosynth-Gene list and 10,027 gene list, resulting in 9,922 overlapped genes. To process PLS analysis, we obtained 3,415 voxels common across intersubject variability of WMFC and 9,922 gene expression maps. Finally, a matrix (3,415 voxels × 9,922 genes) of the brain-wide gene expression for the WMFC was obtained.

6. Enriched disease terms

To explore the potential clinical applications of genes within the top-ranked gene set related to intersubject variability of WMFC, we performed a disease-associations analysis based on WEB-BASED Gene SeT AnaLysis Toolkit [12] (http://webgestalt.org/). We found that genes highly overexpressed in heteromodal regions were primarily enriched for psychiatric diseases (top 10 terms were listed in Supplementary Table 2). However, genes overexpressed in unimodal regions were mostly enriched for neurodegenerative disorders (top 10 terms were listed in Supplementary Table 3).

Supplementary Table 2. Enriched disease terms based on PLS1+ gene list

Gene Set	Description	Size	Overlap	Expect	Enrichment Ratio	P_{FDR}
C0036341	Schizophrenia	956	127	58.34368	2.176757	0
PA447208	Mental Disorders	612	110	37.34972	2.945136	0
PA447216	Schizophrenia	391	78	23.86232	3.268752	0
PA447199	Bipolar Disorder	336	61	20.50573	2.974779	1.50E-11
PA153906318	Autism Spectrum Disorder	238	46	14.52489	3.166977	2.59E-09
C0005586	Bipolar Disorder	488	71	29.78213	2.38398	5.60E-09
PA447278	Depression	215	42	13.12122	3.200921	1.16E-08
PA447209	Mood Disorders	190	39	11.5955	3.363373	1.16E-08
PA444065	Epilepsy	243	45	14.83004	3.034383	1.16E-08
C0004352	Autistic Disorder	267	47	16.29473	2.884368	2.32E-08

Supplementary Table 3. Enriched disease terms based on PLS1– gene list

Gene Set	Description	Size	Overlap	Expect	Enrichment Ratio	P_{FDR}
PA445301	Peripheral Nervous System Diseases	154	32	9.280844	3.447962	3.33E-06
PA446858	Neurodegenerative Diseases	444	59	26.75776	2.204968	1.35E-05
PA445380	Polyneuropathies	128	27	7.713948	3.500153	1.35E-05
PA444939	Metabolism, Inborn Errors	391	54	23.5637	2.29166	1.35E-05
PA443854	Demyelinating Diseases	189	33	11.39013	2.897246	3.55E-05
PA445093	Nervous System Diseases	702	78	42.30619	1.843702	9.78E-05
PA446325	Lysosomal Storage Diseases	82	18	4.941748	3.642436	0.001242
PA446139	Hereditary Motor and Sensory Neuropathies	118	22	7.111296	3.093669	0.001375
PA446788	Muscle Weakness	185	29	11.14907	2.601115	0.001375
PA445108	Neuromuscular Diseases	328	42	19.76699	2.124754	0.001848

7. Differentially expressed genes in multiple sclerosis

In addition, due to multiple sclerosis as a typical WM disease, we also explored the potential clinical relevance of WMFC intersubject variability. We first selected 345 differentially expressed genes-related to multiple sclerosis based on gene expression data from peripheral blood mononuclear cells (Shang Z, et al., 2020), including 196 upregulated genes and 149 downregulated genes. We then obtained these genes' expression maps from Neurosynth-Gene database (https://www.neurosynth.org/genes/), and averaged these maps to obtain one dysregulated map (Supplementary Figure 4a). Finally, we evaluated the spatial correspondence between the dysregulated genes expression and WMFC intersubject variability map on the overlapped voxels. We found that the WMFC intersubject variability was correlated with dysregulated genes expression (r = –0.29, P_{moran} = 0.0002; Supplementary Figure 4b).

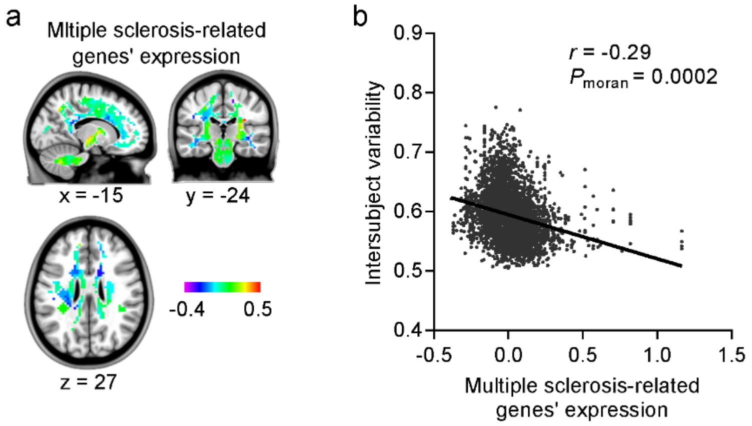

Supplementary Figure 4. Relationship between intersubject variability of WMFC and multiple sclerosis-related genes' expression. a The distribution of multiple sclerosis-related genes' expression. **b** Multiple sclerosis-related genes' expression correlated with WMFC intersubject variability.

8. Local and Long-range WMFC

We explored whether the special network organization of the human brain is related to intersubject variability of WMFC. The degree of distant and local functional connectivity (FC) was quantified at each voxel in the WM. According to previous study [13], distant FC was defined as the connection ($r > 0.25$) between two regions with a distance larger than 25 mm, and local FC was defined as the connection ($r > 0.25$) within 12 mm. We found that the percentage of local FC demonstrated a moderate negative correlation with the intersubject variability of WMFC ($r = –0.24$, $P_{moran} = 0.02$, FDR-corrected; Supplementary Figure 5a) across the WM. However, within the dominated by distant FC, intersubject variability of WMFC was not correlated with the percentage of distant FC ($r = –0.03$, $P_{moran} = 0.43$; Supplementary Figure 5b).

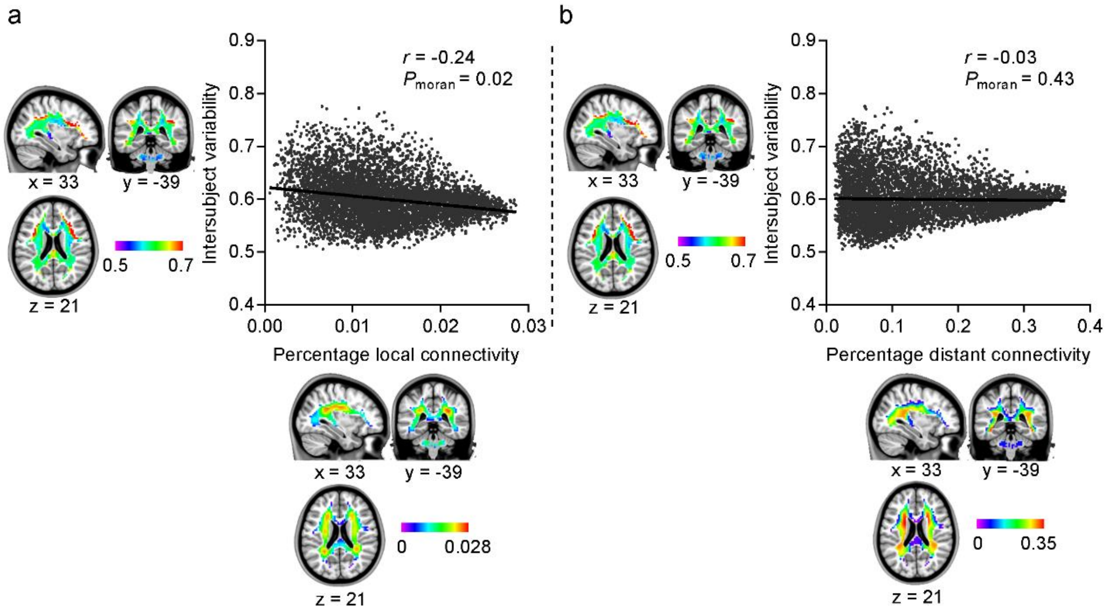

Supplementary Figure 5. Relationship between intersubject variability of WMFC and local and long-range functional connectivity. a Local functional connectivity (FC) negatively correlated with intersubject variability of WMFC. **b** No association between distant FC and intersubject variability of WMFC. *P* values were obtained by MSR method with FDR-corrected. The distributions of percentage of local or distant FC were shown in below.

9. Validation of intersubject variability pattern

To reproduce the intersubject variability, we separately used four sessions. Using a leave-one-out cross-validation scheme, we constructed a PLS regression model [14] based on the first three sessions and tested it on the fourth session. We found that the intersubject variability based on the fourth session was correlated with the predictive intersubject variability based on the first three sessions (r = 0.88, P_{moran}= 0.0001; Supplementary Figure 6).

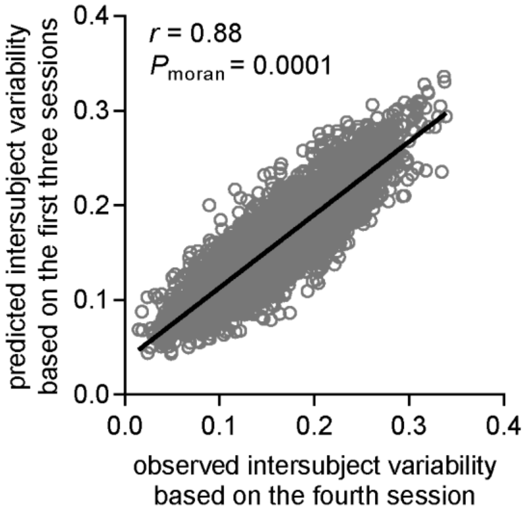

Supplementary Figure 6. Reproducibility of intersubject variability based on the PLS predictive model.

The intersubject variability of WMFC was estimated based on a 3T machine. On that account, we used the four sessions of the resting-state fMRI data from the WU-Minn-Ox HCP 7T dataset [15, 16] (https://db.humanconnectome.org/) to verify the spatial patterns of intersubject variability. The preprocessing steps for 7T images were implemented using the HCP pipeline, which generated all the publicly shared images via the HCP database. These steps have been comprehensively described in the previous HCP publications [17, 18]. Considering that the study focused on WM, we used the preprocessed data of "*Resting State fMRI Functional Preprocessed Extended (59k Mesh + Volume)*" in HCP database. To ensure the same preprocessing steps with this

work, we then regressed out 24 head motion parameters (Friston 24-parameter model), and CSF signals from the resting-state fMRI data. Spatial smoothing was performed with a small Gaussian kernel of 4mm on the volume. Finally, temporal filtering was applied with a band-pass filtering (0.01–0.10 Hz). We excluded the subjects whose who raw data is not complete, and who with mean FD > 0.2mm that is the idientifcal criterion to the main text. Thus, we used resting-state fMRI data of four sessions based on the remained subjects (n = 100). We found that intersubject variability in this work showed similar distribution with intersubject variability based on WU-Minn-Ox HCP 7T dataset across 12 WM functional networks (r = 0.60, P = 0.04; Supplementary Figure 7).

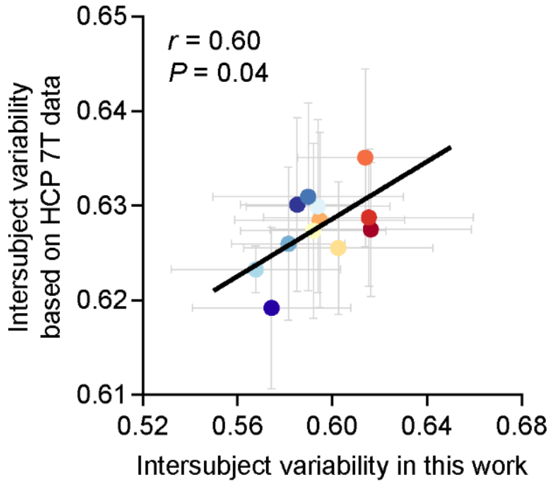

Supplementary Figure 7. Reproducibility of intersubject variability based on the WU-Minn-Ox HCP 7T dataset. Each dot denotes the mean value across voxels within a given WM functional network. The colors represent the WM functional network shown in Figure 2.

10.Shared and specific enrichment pathways between intersubject variability of WMFC and GMFC-related genes

Following demonstrating the intersubject variability of WMFC-related gene annotations, we aimed to explore the distinct and shared transcriptomic underpinnings between WM and GM. The intersubject variability of GMFC was obtained from a previous study [19] (https://balsa.wustl.edu/4mmj1), which was based on Human Connectome Project dataset. For estimation of gene expression in GM, we used the same method for selecting gene list. Because the intersubject variability of GMFC was based on HCPMMP1 comprising 360 regions, 180 per hemisphere, we next assigned samples to regions. To assign samples to brain regions more accurately, the T1-images of six adult brains were first preprocessed using FreeSurfer. The HCPMMP1 employed in the neuroimaging dataset was reconstructed in each AHBA donor brain. Then a threshold was applied to avoid assigning samples beyond a certain distance [20], i.e., samples located less than 2 mm (Euclidean distance) to a region boundary were included. Finally, normalization of expression measures to account for inter-individual differences and outlying values. Gene expression data were normalized using the scaled robust sigmoid [21, 22] for each subject to eliminate the inter-individual differences in expression measures. Excluding the regions without non-expression values, we obtained a 284 (regions) × 10,027 (genes) matrix of the brain-wide gene expression for the GM. After performing PLS analysis, the intersubject variability of GMFC-related genes were obtained.

To facilitate the understanding of pathways (and pathway clusters) that are shared between, or selectively ascribed to, specific gene lists, we performed multi-gene-list meta-analysis [23] between the intersubject variability of WMFC-related and GMFC-related PLS1+ or PLS1– gene list. We found that intersubject variability of WMFC-related and GMFC-related shared almost enrichment pathways in PLS1+ gene lists (Supplementary Figure 8a), and these pathways were related to synapse and glutamate, such as “chemical synaptic transmission”, “synapse organization”, “synaptic

transmission, glutamatergic”, and “Glutamatergic synapse”. However, based on PLS1– gene lists, intersubject variability of WMFC exhibited specific enrichment pathways relative to GMFC, which were dominated by glial-related terms (Supplementary Figure 8b), such as “ensheathment of neurons”, and “regulation of gliogenesis”. The shared and specific transcriptomic underpinnings indicated that intersubject variability of WMFC may provide some complementary information for GMFC to explore brain function.

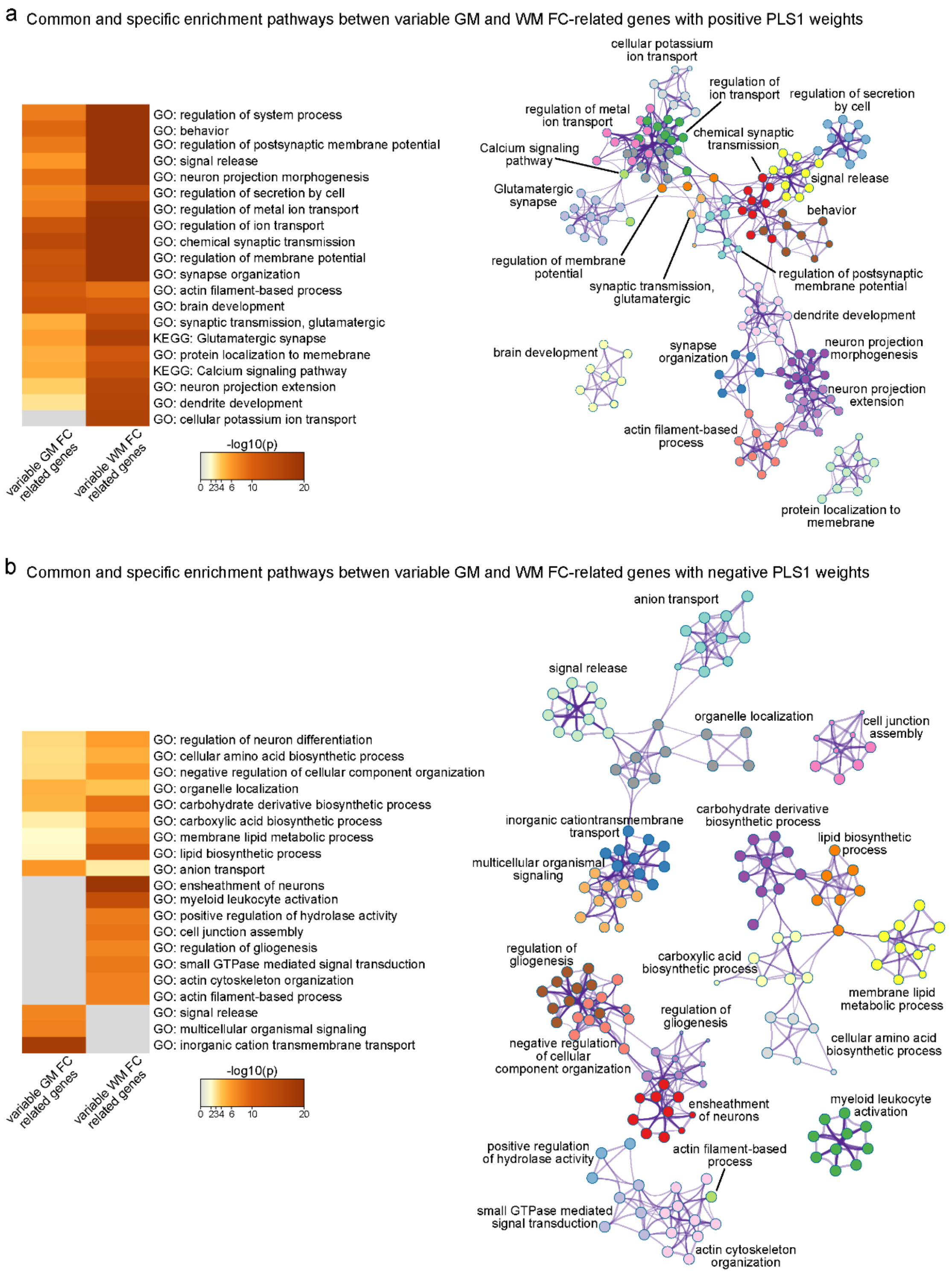

Supplementary Figure 8. Shared and specific transcriptomic underpinnings between intersubject variability of WMFC and GMFC. a For PLS1+ gene lists, almost common enrichment pathways between intersubject variability of WMFC and GMFC. **b** For PLS1– gene lists, specific enrichment pathways between intersubject variability of WMFC and GMFC. All these pathways were corrected by FDR with $p < 0.05$.

Supplementary References

1. Zuo, X. N. et al. An open science resource for establishing reliability and reproducibility in functional connectomics. *Sci Data* **1**, 140049 (2014).
2. Esteban, O. et al. MRIQC: Advancing the automatic prediction of image quality in MRI from unseen sites. *PLoS One* **12**, e0184661 (2017).
3. Esteban, O. et al. Crowdsourced MRI quality metrics and expert quality annotations for training of humans and machines. *Sci Data* **6**, 30 (2019).
4. Laumann, T. O. et al. Functional system and areal organization of a highly sampled individual human brain. *Neuron* **87**, 657-670 (2015).
5. Kong, R. et al. Spatial topography of individual-specific cortical networks predicts human cognition, personality, and emotion. *Cereb Cortex* **29**, 2533-2551 (2019).
6. Hawrylycz, M. J. et al. An anatomically comprehensive atlas of the adult human brain transcriptome. *Nature* **489**, 391-399 (2012).
7. Arnatkeviciute, A., Fulcher, B. D. & Fornito, A. A practical guide to linking brain-wide gene expression and neuroimaging data. *Neuroimage* **189**, 353-367 (2019).
8. Arloth, J. et al. Re-annotator: annotation pipeline for microarray probe sequences. *PLoS One* **10**, e0139516 (2015).
9. Burt, J. B. et al. Hierarchy of transcriptomic specialization across human cortex captured by structural neuroimaging topography. *Nat Neurosci* **21**, 1251-1259 (2018).
10. Richiardi, J. et al. BRAIN NETWORKS. Correlated gene expression supports synchronous activity in brain networks. *Science* **348**, 1241-1244 (2015).
11. Miller, J. A. et al. Transcriptional landscape of the prenatal human brain. *Nature* **508**, 199-206 (2014).
12. Liao, Y. et al. WebGestalt 2019: gene set analysis toolkit with revamped UIs and APIs. *Nucleic Acids Res* **47**, W199-W205 (2019).
13. Mueller, S. et al. Individual variability in functional connectivity architecture of the human brain. *Neuron* **77**, 586-595 (2013).
14. Abdi, H. Partial least squares regression and projection on latent structure regression (PLS Regression). *WIREs Comp Stat* **2**, 97-106 (2010).
15. Elam, J. S. et al. The Human Connectome Project: A retrospective. *Neuroimage* **244**, 118543 (2021).
16. Van Essen, D. C. et al. The WU-Minn Human Connectome Project: an overview. *Neuroimage* **80**, 62-79 (2013).
17. Glasser, M. F. et al. The minimal preprocessing pipelines for the Human Connectome Project. *Neuroimage* **80**, 105-124 (2013).
18. Glasser, M. F. et al. The Human Connectome Project's neuroimaging approach. *Nat Neurosci* **19**, 1175-1187 (2016).
19. Demirtas, M. et al. Hierarchical heterogeneity across human cortex shapes large-scale neural dynamics. *Neuron* **101**, 1181-1194 e1113 (2019).
20. Romero-Garcia, R. et al. Structural covariance networks are coupled to expression of genes enriched in supragranular layers of the human cortex. *Neuroimage* **171**, 256-267 (2018).

21. Fulcher, B. D., Little, M. A. & Jones, N. S. Highly comparative time-series analysis: the empirical structure of time series and their methods. *J R Soc Interface* **10**, 20130048 (2013).
22. Fulcher, B. D. & Fornito, A. A transcriptional signature of hub connectivity in the mouse connectome. *Proc Natl Acad Sci U S A* **113**, 1435-1440 (2016).
23. Zhou, Y. et al. Metascape provides a biologist-oriented resource for the analysis of systems-level datasets. *Nat Commun* **10**, 1523 (2019).
