# Supplementary material for: Transcriptomic and macroscopic architectures of intersubject functional variability in human brain white-matter
Source: Commun Biol. 2021 Dec 20;4:1417. doi: 10.1038/s42003-021-02952-y (PMC8688465; doi:10.1038/s42003-021-02952-y)
Supplement: Supplementary file 2 — Description of Additional Supplementary Files [file 42003_2021_2952_MOESM2_ESM.pdf]

Description of Additional Supplementary Files

File name: Supplementary Data 1
Description: Source Data.
