# Supplementary material for: Transcriptomic and macroscopic architectures of intersubject functional variability in human brain white-matter
Source: Commun Biol. 2021 Dec 20;4:1417. doi: 10.1038/s42003-021-02952-y (PMC8688465; doi:10.1038/s42003-021-02952-y)
Supplement: Supplementary file 4 — Reporting Summary [file 42003_2021_2952_MOESM4_ESM.pdf]

nature research

Corresponding author(s): Huafu CHEN; Wei LIAO

Last updated by author(s): Nov 18, 2021

Reporting Summary

Nature Research wishes to improve the reproducibility of the work that we publish. This form provides structure for consistency and transparency in reporting. For further information on Nature Research policies, see our Editorial Policies and the Editorial Policy Checklist.

Statistics

For all statistical analyses, confirm that the following items are present in the figure legend, table legend, main text, or Methods section.

n/a	Confirmed	
☐	☒	The exact sample size (n) for each experimental group/condition, given as a discrete number and unit of measurement
☐	☒	A statement on whether measurements were taken from distinct samples or whether the same sample was measured repeatedly
☐	☒	The statistical test(s) used AND whether they are one- or two-sided<br>*Only common tests should be described solely by name; describe more complex techniques in the Methods section.*
☐	☒	A description of all covariates tested
☐	☒	A description of any assumptions or corrections, such as tests of normality and adjustment for multiple comparisons
☐	☒	A full description of the statistical parameters including central tendency (e.g. means) or other basic estimates (e.g. regression coefficient) AND variation (e.g. standard deviation) or associated estimates of uncertainty (e.g. confidence intervals)
☐	☒	For null hypothesis testing, the test statistic (e.g. F, t, r) with confidence intervals, effect sizes, degrees of freedom and P value noted<br>*Give P values as exact values whenever suitable.*
☒	☐	For Bayesian analysis, information on the choice of priors and Markov chain Monte Carlo settings
☒	☐	For hierarchical and complex designs, identification of the appropriate level for tests and full reporting of outcomes
☐	☒	Estimates of effect sizes (e.g. Cohen's d, Pearson's r), indicating how they were calculated

Our web collection on statistics for biologists contains articles on many of the points above.

Software and code

Policy information about availability of computer code

Data collection	Multiple MRI parameters (see Methods)
Data analysis	Both BOLD-fMRI and ASL images were preprocessed using the DPARSF (v4.3, www.restfmri.net) and SPM12 (https://www.fil.ion.ucl.ac.uk/spm/software/spm12/).<br>The DWI data were preprocessed on volumetric space using FSL (v6.0, https://fsl.fmrib.ox.ac.uk/fsl/fslwiki).<br>The T1w/T2w ratio was calculated using MRTOOL (https://www.nitrc.org/projects/mrtool/).<br>The code for gene expression analysis can be found at https://github.com/BMHLab/AHBAprocessing.<br>Gene enrichments were analyzed at https://metascape.org/gp/index.html#/main/step1.<br>The code for spatial permutation testing was implemented in BrainSpace (https://www.brainspace.com/).<br>Disease-association terms were obtained from WebGestalt website (http://www.webgestalt.org/).<br>The 7T Human connectome dataset is available at https://db.humanconnectome.org/.

For manuscripts utilizing custom algorithms or software that are central to the research but not yet described in published literature, software must be made available to editors and reviewers. We strongly encourage code deposition in a community repository (e.g. GitHub). See the Nature Research guidelines for submitting code & software for further information.

Data

Policy information about availability of data

All manuscripts must include a data availability statement. This statement should provide the following information, where applicable:

- Accession codes, unique identifiers, or web links for publicly available datasets
- A list of figures that have associated raw data
- A description of any restrictions on data availability

Human gene expression maps that support the findings of this study are available Neurosynth-Gene database (https://www.neurosynth.org/genes/) based on the Allen Brain Atlas (https://human.brain-map.org/static/download).
Compiled cell-specific gene set list from all available large-scale single-cell studies of the adult human cortex can be obtained from the raw Seidlitz et al.39 dataset (https://static-content.springer.com/esm/art%3A10.1038%2Fs41467-020-17051-5/MediaObjects/41467_2020_17051_MOESM8_ESM.xlsx).
The code for preprocessing of WM functional data can be found at https://github.com/weiliao81/WMFPrep.

Field-specific reporting

Please select the one below that is the best fit for your research. If you are not sure, read the appropriate sections before making your selection.

☒ Life sciences ☐ Behavioural & social sciences ☐ Ecological, evolutionary & environmental sciences

For a reference copy of the document with all sections, see nature.com/documents/nr-reporting-summary-flat.pdf

Life sciences study design

All studies must disclose on these points even when the disclosure is negative.

Sample size	Sample size was based on availability of data, no statistical methods were used to pre-determine sample size.
Data exclusions	Subjects were excluded if they: had no history of neurologic or psychiatric conditions, and no gross abnormalities on brain MRI. In addition, data were excluded (mean framewise displacement > 0.2mm) based on the quality of the functional MRI scans .
Replication	Several sensitivity analyses assessed robustness. In addition, we used 7T HCP database to assess intersubject variability of white-matter (WM) functional connectivity, and found the similar distribution between the two data sets across 12 WM functional networks.
Randomization	To robustly estimate p values, we employ multiple randomization (permutation) strategies and taking into account potential confounding effects of spatial autocorrelation.
Blinding	Blinding was not relevant to our study. We enrolled healthy subjects in our study.

Reporting for specific materials, systems and methods

We require information from authors about some types of materials, experimental systems and methods used in many studies. Here, indicate whether each material, system or method listed is relevant to your study. If you are not sure if a list item applies to your research, read the appropriate section before selecting a response.

Materials & experimental systems

n/a	Involved in the study	
☒	☐	Antibodies
☒	☐	Eukaryotic cell lines
☒	☐	Palaeontology and archaeology
☒	☐	Animals and other organisms
☐	☒	Human research participants
☒	☐	Clinical data
☒	☐	Dual use research of concern

Methods

n/a	Involved in the study	
☒	☐	ChIP-seq
☒	☐	Flow cytometry
☐	☒	MRI-based neuroimaging

Human research participants

Policy information about studies involving human research participants

Population characteristics	All information can be found in Methods section.
Recruitment	Subjects were recruited from the University of Electronic Science and Technology of China, China.

Ethics oversight	This longitudinal study was approved by the Local Medical Ethics Committee of the University of Electronic Science and Technology of China (UESTC), China. Written informed consent was obtained from all subjects prior to scanning.

Note that full information on the approval of the study protocol must also be provided in the manuscript.

Magnetic resonance imaging

Experimental design

Design type	Multi-MRI acquisition
Design specifications	4 scans per participant
Behavioral performance measures	NA

Acquisition

Imaging type(s)	Structural, resting-state fMRI, and diffusion
Field strength	3 Tesla
Sequence & imaging parameters	See Methods for detailed information
Area of acquisition	Whole brain scan

Diffusion MRI ☒ Used ☐ Not used

Parameters	60 non-collinear directions b = 1,000 s/mm2.

Preprocessing

Preprocessing software	Both BOLD-fMRI and ASL images were preprocessed using the DPARSF (v4.3, http://rfmri.org/DPARSF) and SPM12 (https://www.fil.ion.ucl.ac.uk/spm/software/spm12/).<br>The DWI data were preprocessed on volumetric space using FSL (v6.0, https://fsl.fmrib.ox.ac.uk/fsl/fslwiki).<br>The T1w/T2w ratio was calculated for the same subject using MRTOOL (https://www.nitrc.org/projects/mrtool/).
Normalization	The neuroimaging data were normalized into the standard Montreal Neurological Institute space.
Normalization template	MNI152
Noise and artifact removal	Diffusion-weighted images were corrected for eddy current distortions and head motion using the FMRIB Software Library. For the rs-fMRI data, the first 5 images were excluded to ensure steady-state signal equilibrium. And then, fMRI images were corrected by realignment, slice timing, detrend, and band-pass filtering (0.01-0.1Hz). We also corrected for head motion and cerebrospinal fluid signals.
Volume censoring	DPARSF for the fMRI analysis.

Statistical modeling & inference

Model type and settings	General linear model was used for each voxel across subjects to determine intersubject variability values (See Methods). These continuous intersubject values were carried forward in the neuroimaging-transcriptomic analyses, where multivariate methods (PLS regression) was performed.
Effect(s) tested	See previous point.

Specify type of analysis: ☒ Whole brain ☐ ROI-based ☐ Both

Statistic type for inference (See Eklund et al. 2016)	Whole-brain continuous values were carried forward to neuroimaging-transcriptomic analyses.
Correction	See Methods and above for descriptions on the multiple permutation strategies employed to test robustness of empirical effects.

Models & analysis

n/a	Involved in the study
☐	☒ Functional and/or effective connectivity
☒	☐ Graph analysis
☐	☒ Multivariate modeling or predictive analysis

Functional and/or effective connectivity	Pearson's correlation
Multivariate modeling and predictive analysis	Partial least squares regression was used relate the intersubject variability values to the post mortem gene expression measurements for all backgroup genes, and to rank genes based on their spatial relationship to variability map.
